# Watson-Crick Base-Pairing Requirements for ssDNA Recognition and Processing in Replication-Initiating HUH Endonucleases

**DOI:** 10.1128/mbio.02587-22

**Published:** 2022-12-21

**Authors:** Adam T. Smiley, Kassidy J. Tompkins, Matthew R. Pawlak, August J. Krueger, Robert L. Evans, Ke Shi, Hideki Aihara, Wendy R. Gordon

**Affiliations:** a Department of Biochemistry, Molecular Biology, and Biophysics, University of Minnesota, Minneapolis, Minnesota, USA; Oregon State University

**Keywords:** HUH endonuclease, HUH-seq, next-generation sequencing, origin of replication, Rep, Watson-Crick base pair, covalent adduct, protein DNA bioconjugation

## Abstract

Replication-initiating HUH endonucleases (Reps) are sequence-specific nucleases that cleave and rejoin single-stranded DNA (ssDNA) during rolling-circle replication. These functions are mediated by covalent linkage of the Rep to its substrate post cleavage. Here, we describe the structures of the endonuclease domain from the Muscovy duck circovirus Rep in complex with its cognate ssDNA 10-mer with and without manganese in the active site. Structural and functional analyses demonstrate that divalent cations play both catalytic and structural roles in Reps by polarizing and positioning their substrate. Further structural comparisons highlight the importance of an intramolecular substrate Watson-Crick (WC) base pairing between the −4 and +1 positions. Subsequent kinetic and functional analyses demonstrate a functional dependency on WC base pairing between these positions regardless of the pair’s identity (i.e., A·T, T·A, G·C, or C·G), highlighting a structural specificity for substrate interaction. Finally, considering how well WC swaps were tolerated *in vitro*, we sought to determine to what extent the canonical −4T·+1A pairing is conserved in circular Rep-encoding single-stranded DNA viruses and found evidence of noncanonical pairings in a minority of these genomes. Altogether, our data suggest that substrate intramolecular WC base pairing is a universal requirement for separation and reunion of ssDNA in Reps.

## INTRODUCTION

**FIG 1:**
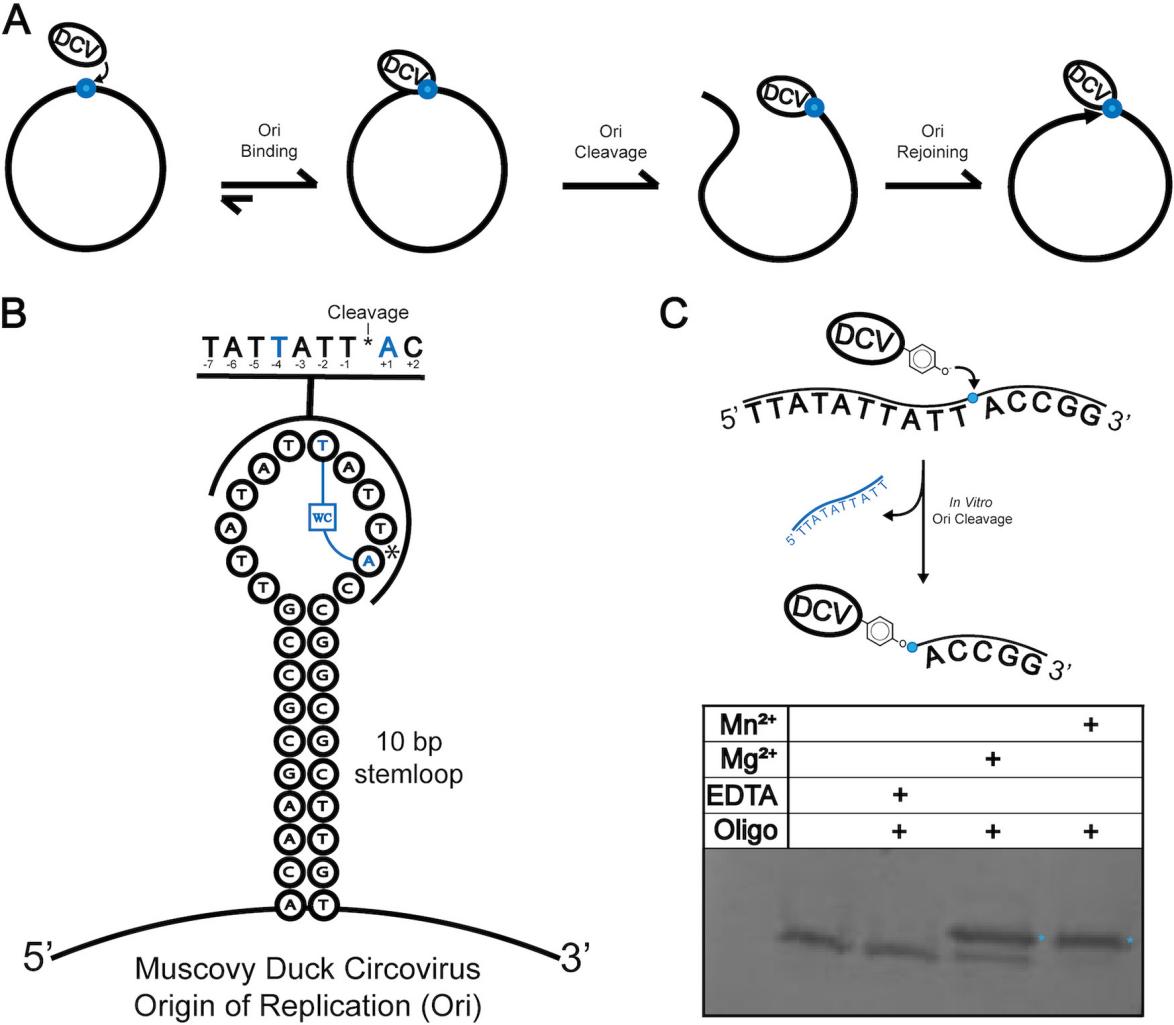
Cartoon depictions of DCV Rep function. (A) Graphical depiction of the Rep-mediated steps in viral genome manipulation characterized throughout the manuscript. Replication occurs post cleavage and prior to rejoining. (B) Cartoon representation of the hairpin loop that the Ori and its flanking sequences form in the DCV genome. The specific nonanucleotide cleavage motif is highlighted, the cleavage site is represented by an asterisk, and the base pair-forming nucleotides are colored cyan. (C) Schematic of an *in vitro* HUH endonuclease cleavage reaction using recombinantly expressed DCV Rep and synthetic ssDNA oligonucleotides derived from the cognate Ori sequence. At the bottom is an image of an SDS-PAGE gel demonstrating cleavage activity across a number of conditions as indicated by an apparently higher-molecular-weight band (cyan asterisk) indicative of the formation of a covalent adduct between the DCV Rep and its substrate.

HUH endonucleases are diverse enzymes that contain an eponymous motif consisting of a pair of conserved metal-coordinating histidine (H) separated by a bulky hydrophobic residue (U) (1). Replication initiator proteins (Reps) from this HUH endonuclease superfamily are found in a variety of bacterial plasmids and viral genomes, including circular Rep-encoding single-stranded DNA (CRESS-DNA) viruses that are known to infect organisms across all three domains of life (1–5). These enzymes initiate rolling-circle replication through sequence-specific processing of the viral single-stranded DNA (ssDNA) genome via cleavage and subsequent phosphotyrosine covalent linkage to the ssDNA’s newly exposed 5′ end (1, 6, 7). Following linkage, the genome’s freed 3′-OH can be used as a primer for DNA replication before its ultimate return to the active site, where it attacks the phosphotyrosine intermediate to rejoin and release the circular ssDNA genome (Fig. 1A) (1). Dual histidine-mediated divalent cation coordination and catalytic tyrosine cleavage/covalent linkage are universal among the HUH endonuclease superfamily, which consists of the previously mentioned Reps, relaxases involved in bacterial conjugation, and DNA transposases, which include the eukaryotic Helitron rolling-circle transposases and the prokaryotic insertion sequence transposase families (1, 4, 8–11).

HUH endonucleases have recently been deployed in a wide variety of biotechnology applications as functional fusion proteins, or “HUH-tags” (6, 7). For example, these nucleases have been applied in CRISPR-Cas9 genome engineering as a means by which to directly tether an ssDNA repair template to Cas9 in order to enhance homology-directed repair (12) and in receptor-specific cell targeting by adeno-associated viruses displaying HUH-tags for covalent linkage of oligonucleotide-conjugated guiding antibodies (13). HUH-tags have proven to be amenable to these applications due to their compact size and their ability to quickly and specifically form robust and nonlabile linkages with ssDNA. Moreover, these nucleases are critical components in the mediation of viral infection across all domains of life, such as crop-ravaging geminiviruses (14) and medically relevant parvoviruses, like B19 (15). Reps are also important tools in gene therapy, as they are responsible for catalyzing the integration of genetic payloads into a host genome via adeno-associated virus-mediated transduction (16). Thus, it is critical to understand the molecular underpinnings of ssDNA processing by viral Reps to identify the key factors of both the protein and its DNA target for recognition, sequence specificity, binding, cleavage, and reversal.

The first cocrystal structures of viral Reps in complex with their specific substrates provided insight into their mechanisms of DNA recognition (6). Strikingly, the ssDNA conforms into a distinct U shape around a key topologically stabilizing motif in the Rep called the single-stranded DNA bridging motif (sDBM). Previous investigations into the structure-function relationship of viral Reps and their cognate substrates revealed that the sDBM motif is chiefly responsible for sequence specificity in these enzymes and that rational swaps of this motif between distantly related Reps can predictably modulate sequence specificity (6). The U-shape reinforced by this motif is reminiscent of the hairpin loop that the cognate origin of replication (Ori) forms within a CRESS-DNA viral genome and is seemingly bolstered by intramolecular hydrogen bonding and a single Watson-Crick (WC) base pair (Fig. 1B). Many nucleic acid-interacting enzymes require their substrates to be structured or bent into specific, and sometimes subtle, conformations for nucleolytic action to occur (17–21). While the WC base pair is conserved among the few viral Rep-ssDNA cocomplexes solved to date (6, 22), the importance of this substrate interaction in binding, cleavage, and reunion is unknown. Moreover, a more comprehensive understanding of the substrate conformational requirements for function in Reps is necessary for furthering their development as highly specific and orthogonal bioconjugation tools.

To further understand the putative roles that ssDNA intramolecular interactions play in conformational priming of the substrate for Rep function, we have conducted a series of structural and functional analyses on the endonuclease domain derived from the Muscovy duck circovirus Rep (DCV). By solving and comparing two DCV-ssDNA cocrystal structures with and without manganese coordinated in the active site, we see that the substrate undergoes repositioning in the presence of Mn^2+^. This indicates that the ion prompts the DNA backbone to conform to a position more conducive to cleavage and suggests that divalent cations play both a catalytic and structural role in Reps, which is seen broadly in a range of nucleolytic enzymes (17–21). Interestingly, the substrate WC base pair is retained across both structures, suggesting that this interaction is mediated by the enzyme independently of coordinated cations. We go on to demonstrate that this WC base pair contributes dramatically to DCV-mediated ssDNA binding, cleavage, and reunion and that its absence nearly always ablates function. Additional interrogation revealed that substrate WC base pair swaps are generally well tolerated, expanding the sequence space that can be harnessed in efforts to engineer novel sequence specificity in Reps. Finally, we investigated how conserved the cognate −4T·+1A WC base pair is across the Ori sequences of all available CRESS-DNA viral genomes and found evidence that a minority of these sequences contain a pair of concomitant mutations that swap the cognate WC with a noncognate WC base pairing, further highlighting the seemingly universal requirements of this substrate intramolecular interaction in these enzymes.

## RESULTS

### Viral Reps bind short cognate ssDNA sequences with high affinity in cooperation with divalent cations.

In order to better understand the Rep-ssDNA interaction, we first aimed to characterize the affinity that the viral DCV Rep has for its ssDNA substrate. Fluorescence polarization (FP) has been used previously to demonstrate that the closely related relaxase HUH endonucleases bind their cognate DNA sequences with subnanomolar affinity (23). However, relaxases have an extended DNA binding interface in comparison to viral Reps, which includes the seemingly distal binding to a hairpin structure 5′ of the Ori sequence that likely contributes to this high affinity. Thus, we developed a fluorescence polarization assay to measure binding affinities of DCV to its Ori sequence. A catalytically deficient DCV Rep mutant (dDCV), in which the bond-forming tyrosine was mutated to phenylalanine (Y91F), was titrated into a fixed concentration of a 3′-end fluorescein (FAM)-labeled synthetic oligonucleotide derived from the Rep’s cognate Ori sequence. Increases in fluorescence anisotropy/polarization are indicative of protein-DNA binding. Due to the modest molecular weight of most Rep proteins (often less than 15 kDa), we constructed a maltose binding protein (MBP) N-terminal fusion of dDCV (MBP-dDCV) (see Fig. S1 in the supplemental material) in order to increase the change in anisotropy amplitude; MBP-dDCV was used in all FP experiments unless otherwise noted. We first calculated dissociation constants (*K_D_*) of dDCV Rep-Ori binding under a range of divalent ion and salt concentrations in order to characterize their effects on dDCV Rep-nucleic acid interactions. It is well-known that HUH endonucleases require divalent metals for catalysis, and, indeed, Rep cleavage/covalent linkage is inhibited in the presence of EDTA (Fig. 1C). As such, we wondered to what extent these divalent metals participate in the noncatalytic aspects of Rep-nucleic acid interaction, such as the substrate binding and conformational priming.

10.1128/mbio.02587-22.1 FIG S1 (A) SDS-PAGE analysis of 5 μg purified, recombinant MBP-dDCV protein (theoretical molecular weight [MW], 54 kDa). (B) Standard size exclusion (SEC) calibration and analytical SEC analysis of 100 μL of 13 mg/mL (250 μM) MBP-dDCV confirm the protein remains as a monomer even in the presence of 5 mM MgCl_2_ at high protein concentrations. (C) Fluorescence polarization/anisotropy comparison between dDCV and MBP-dDCV demonstrating that while there is a modest change in calculated dissociation constants, the amplitude of signal is greatly improved in the context of the higher-molecular-weight fusion protein. (D) *In vitro* HUH reaction of substrates used in wc- and rwcHUH-seq reactions performed in 50 mM NaCl, 50 mM HEPES, pH 8.0, 1 mM cation, 1 mM DTT, 3 μM SUMO-DCV, and 3 μM oligonucleotide substrate at ambient temperature for 1 hour. Reactions were then run on an SDS-PAGE gel and Coomassie stained for visualization of covalent linkage. Apparent increases in molecular weight are the result of covalent conjugation of the oligonucleotide to DCV Rep. Lanes 1 to 4 and 8 to 11 are reactions with rwcHUH-seq preconjugation substrates in the presence of magnesium and manganese, respectively. Lanes 5, 6, 12, and 13 are reactions with the up- and downstream constant regions of the wcHUH-seq library oligonucleotide. Lanes 7 and 14 are reactions with the wcHUH-seq library. Download FIG S1, TIF file, 2.0 MB. Copyright © 2022 Smiley et al. 2022 Smiley et al. https://creativecommons.org/licenses/by/4.0/ This content is distributed under the terms of the Creative Commons Attribution 4.0 International license.

**FIG 2:**
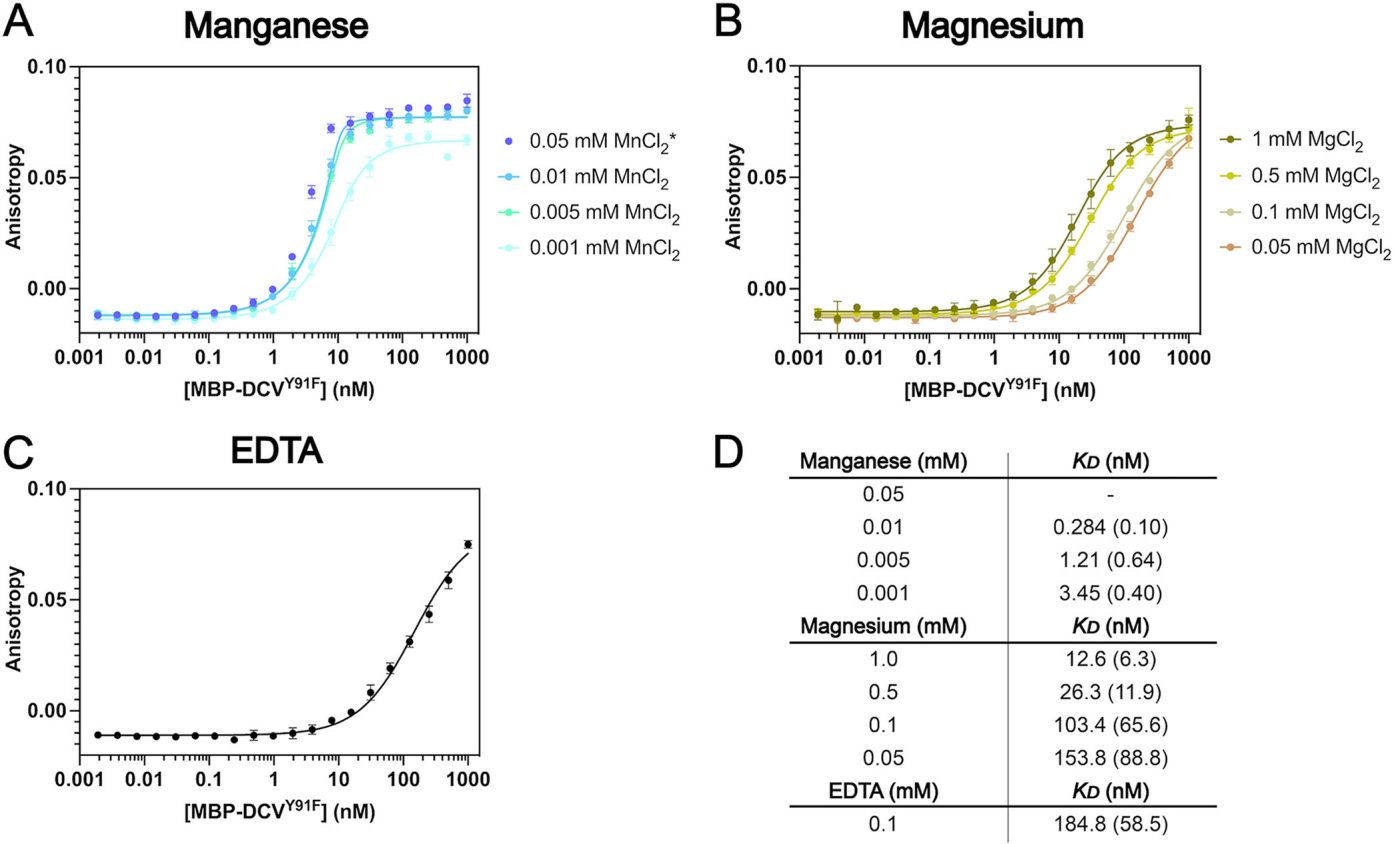
Cation-dependent differences in dDCV Rep substrate binding. Fluorescence anisotropy affinity titrations using 10 nM D*-ori*-F oligonucleotide and MBP-DCV^Y91F^ (MBP-dDCV) Rep at various concentrations of divalent cations. (A) Binding curves across a range of manganese concentrations. The asterisk indicates the data could not be fit to the *K_D_* calculation. (B) Binding curves across a range of magnesium concentrations. (C) Binding curves in the presence of 0.1 mM EDTA (i.e., no cation). (D) Mean dissociation constants calculated across each condition in triplicate. Standard deviation is in parentheses.

In the presence of saturating Mg^2+^, the *K_D_* was 12.6 nM, while in the presence of saturating Mn^2+^, the *K_D_* was too low to be measured by this assay (Fig. 2A, B, and D). Thus, we titrated the metal across a range of lower-than-saturating concentrations in an attempt to characterize the *K_D_* of DNA for DCV in the presence of Mn^2+^ and approximated it to be <0.1 nM (Fig. 2D). In the presence of EDTA, the binding affinity was measured to be 150 nM, indicating that binding can occur in the absence of metal (Fig. 2C and D). Furthermore, Ca^2+^ has a very minimal positive effect on affinity in comparison to EDTA, corroborating the fact that this cation cannot catalyze substrate transesterification in other HUH endonucleases (Table S1). Increasing concentrations of NaCl have a strong and predictable negative correlation with *K_D_* in the presence of both Mn^2+^ and Mg^2+^ (Table S1). Altogether, DCV Rep binding affinity to cognate DNA is strongly dependent on the type of ionic cofactor, the concentration of the ionic cofactor, and the ionic strength (i.e., the concentration of NaCl), where the lowest observed *K_D_* occurs at high concentrations of Mn^2+^ and low concentrations of NaCl. The dramatically enhanced binding affinity that DCV Rep has for its DNA substrate in the presence of divalent cation suggests the metal is playing additional noncatalytic roles in Rep-ssDNA interaction.

10.1128/mbio.02587-22.4 TABLE S1 *K_D_* values reported for fluorescence anisotropy affinity titrations using 10 nM D-*ori*-F oligonucleotide and MBP-DCV^Y91F^ (MBP-dDCV) Rep at various concentrations of divalent cations and NaCl. Each assay was performed in quadruplicate either one or two times. Download Table S1, DOCX file, 0.01 MB. Copyright © 2022 Smiley et al. 2022 Smiley et al. https://creativecommons.org/licenses/by/4.0/ This content is distributed under the terms of the Creative Commons Attribution 4.0 International license.

### Divalent cations have dual structural and catalytic functions.

We previously obtained cocrystals structures of the nuclease domain of two Reps derived from porcine circovirus 2 (PCV2; PDB accession no. 6WDZ) and wheat dwarf virus (WDV; PDB accession no. 6WE0) bound to their respective cognate Ori sequences, revealing the cooperative inter- and intramolecular factors responsible for ssDNA binding and recognition (6). Each of these structures contained a coordinated manganese positioned in the active site by the HUH (or HUQ in circovirus Reps) motif. To better understand the roles that coordinated divalent ions play in Rep function, we resolved two cocrystal structures of the endonuclease domain of DCV Rep in complex with a cognate ssDNA 10-mer (5′-TATTATTACC-3′) derived from the DCV Ori, with and without manganese coordinated in the active site. The DCV Rep structure with Mn^2+^ bound resolved to 1.30 Å (PDB accession no. 7KII), and the structure absent of Mn^2+^ resolved to 1.69 Å (PDB accession no. 7KIJ) (Table S2). Precleavage complexes were obtained using the dDCV mutant. The substrate in both structures conforms to the characteristic “U”-shaped architecture, indicating that the ssDNA conformation is largely facilitated by the DNA binding interface and intramolecular base pairing and base stacking, with little support from the coordinated cation.

**FIG 3:**
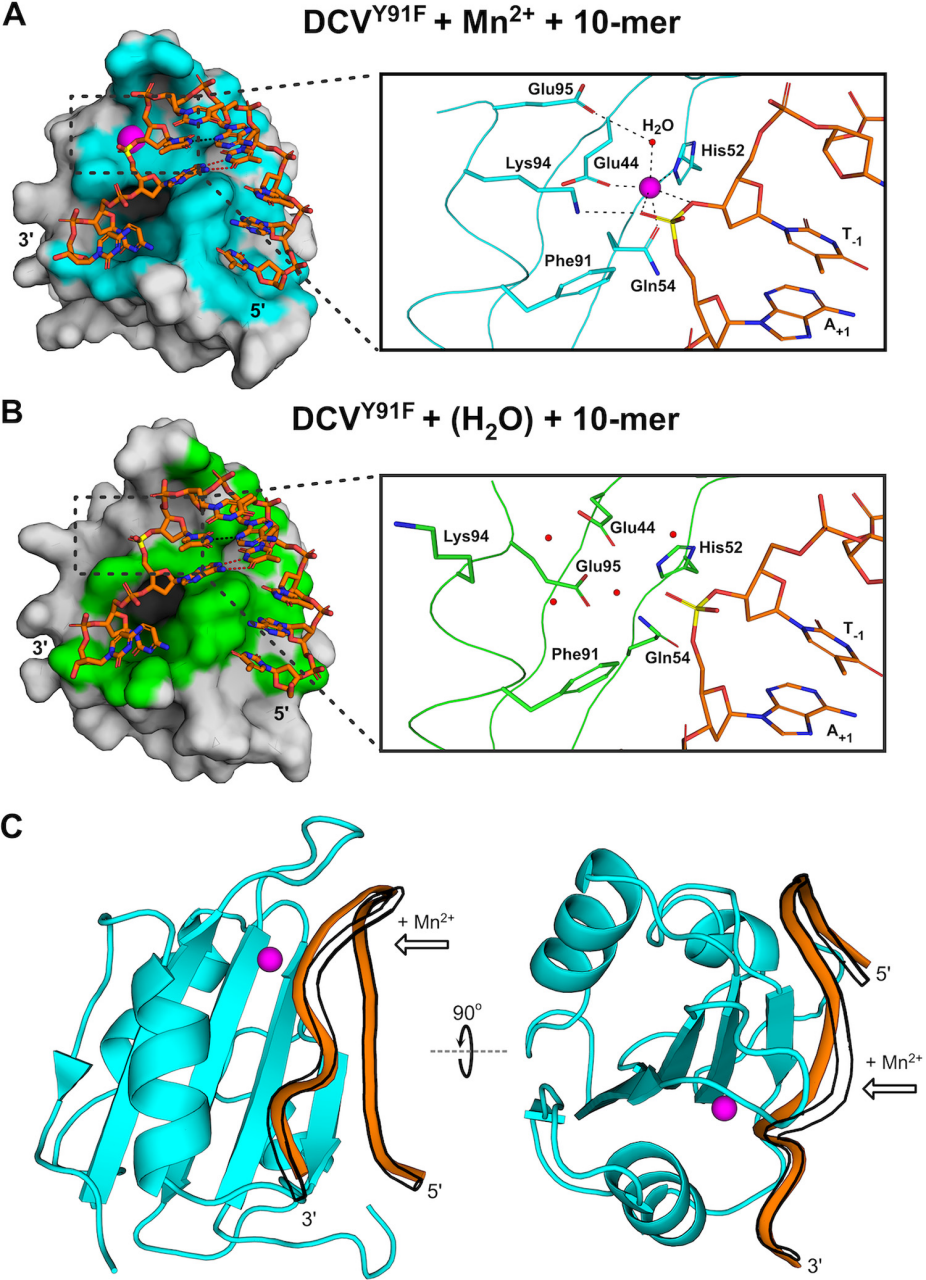
Overall cocrystal structure(s) of DCV Rep bound to its cognate ssDNA substrate. (A and B) Cocrystal structures of DCV Rep with a coordinated manganese in its active site (PDB accession no. 7KII) (A) and without the ion (PDB accession no. 7KIJ) (B) in complex with a cognate ssDNA 10-mer. DCV Reps are displayed as a surface representation in gray with atoms within 4 Å of DNA highlighted in either cyan (+Mn^2+^) or green (−Mn^2+^). The ssDNAs displayed as sticks are colored orange by element with the scissile phosphate highlighted in yellow. DNA conforms to a U-shaped architecture facilitated by the WC base pair (red dashes). The zoom panels show an active site comparison of DCV Rep bound with and without the metal ion. A triad of residues, Glu44, His52, and Gln54, along with water (red spheres) and the scissile phosphate coordinate the manganese. In the absence of the ion, four waters occupy the space at the center of the active site between the coordination triad. (C) Structural cartoon representation of DCV (cyan) bound to ssDNA backbone (orange) and manganese (magenta). DCV bound to ssDNA without manganese was superimposed onto the DCV structure with manganese. Only the ssDNA backbone, represented as a transparent cartoon outlined in black, is shown to contrast the position of the ssDNA backbone between the metal ion- and nonmetal ion-bound states. A white arrow indicates positions between the two structures with the most dramatic changes in position across the conditions.

In the DCV plus Mn^2+^ structure, the Mn^2+^ is coordinated by His52 and Gln54 within the HUQ motif along with the third residue of the coordinating triad Glu44, and a water, which, in turn, is positioned by Glu95 (Fig. 3A). Two hydrogen bonds from the scissile phosphate complete the octahedral coordination of Mn^2+^. Interestingly, four waters occupy the active site in the absence of Mn^2+^ coordination, and the ion-coordinating triad side chains shift slightly away from the direction of the space occupied by the cation in the Mn^2+^-bound structure (Fig. 3B). A conserved Lys94 is oriented toward the catalytic residue and facilities a hydrogen bond with the scissile phosphate, adding evidence that this lysine may not only be a general base but that it might also play a cooperative structural role with the ion in stabilizing the ssDNA backbone in the active site. Indeed, Lys94 is positioned away from the active site in the absence of Mn^2+^ coordination (Fig. 3B).

In further comparisons of these two structures, we noticed the ssDNA substrate seemed to be positioned much farther into the active site in the presence of Mn^2+^ (Fig. 3C). Given this apparent conformational rearrangement in the presence of cation, we explored how the metal affects overall ssDNA binding and conformation from a structural perspective. Using DNAproDB (24, 25), we calculated the number of polar and nonpolar contacts between ssDNA and the Rep as well as the buried solvent-accessible surface area (BASA) at each nucleotide (nt) position (Fig. S2A and B). Unsurprisingly, due to the overall conformational similarity of the ssDNA between the two DCV Rep structures, there are very few differences between the number of contacts within 4 Å at each nucleotide facilitated by the Rep and BASA values. However, we observed a notable loss of 19 total contacts (4 of which are hydrogen bonds) at position T_−1_ when Mn^2+^ is not present, indicating a dynamic localized conformational shift in that region (Fig. S2B). A significant loss of contacts is also seen at position T_−8_; however, this is due to contacts from Ser4 and Tyr7 within an unstructured region of the N terminus that is only present in the Mn^2+^-bound structure, indicating these are nonspecific, transient interactions (Fig. S2B). We further probed differences in these structures through modeling a Tyr into position Phe91 in both structures to measure the distance between the oxygen of the phenol and the scissile phosphate. In the absence of Mn^2+^, the measured distance is 3.0 Å, whereas when Mn^2+^ is coordinated in the active site, and the scissile phosphate is pulled 0.7 Å closer to the oxygen, with a measured distance of 2.3 Å (Fig. S2D). Considering that Mn^2+^ coordination includes two ion-dipole bonds with the scissile phosphate, it is likely this is the driving force behind bringing the tyrosine in close proximity to the phosphate.

10.1128/mbio.02587-22.2 FIG S2 (A) Schematic of DNproDB analysis of DCV structures in complex with ssDNA with and without manganese bound in the active site. The far 5′ (position −7) end of the substrates is in the bottom right section of the U shape in the graphics, and the far 3′ (position +3) is in the bottom left. Cleavage occurs between the −1T and the +1A. (B) Summary of BASA of ssDNA and number of protein-ssDNA contacts in DCV structures with and without manganese present in the active site. (C) Simulated annealed omit maps (m*F*_o_-D*F*_c_) of DCV Rep active sites displayed as a blue mesh contoured at σ of 2 or 3 show strong evidence for coordinated Mn^2+^ in the active site, while there is moderate support for four uncoordinated waters in the active site when Mn^2+^ is not bound. (D) The scissile phosphate shifts 0.7 Å closer to the oxygen of modeled tyrosine 91 residue when Mn^2+^ is coordinated in the active site. Download FIG S2, TIF file, 2.7 MB. Copyright © 2022 Smiley et al. 2022 Smiley et al. https://creativecommons.org/licenses/by/4.0/ This content is distributed under the terms of the Creative Commons Attribution 4.0 International license.

Overall, the two structures are remarkably similar, particularly in substrate architecture. The bound ssDNA retains its stabilizing intramolecular WC base pair and base stacking regardless of ion coordination. This suggests that while the ion does play an important role in both substrate polarization and positioning, it has little impact on its conformation, which is largely enforced by the nucleic acid’s intramolecular interactions and contacts with the Rep’s sDBM.

### The substrate WC base pair is critical for DNA binding.

**FIG 4:**
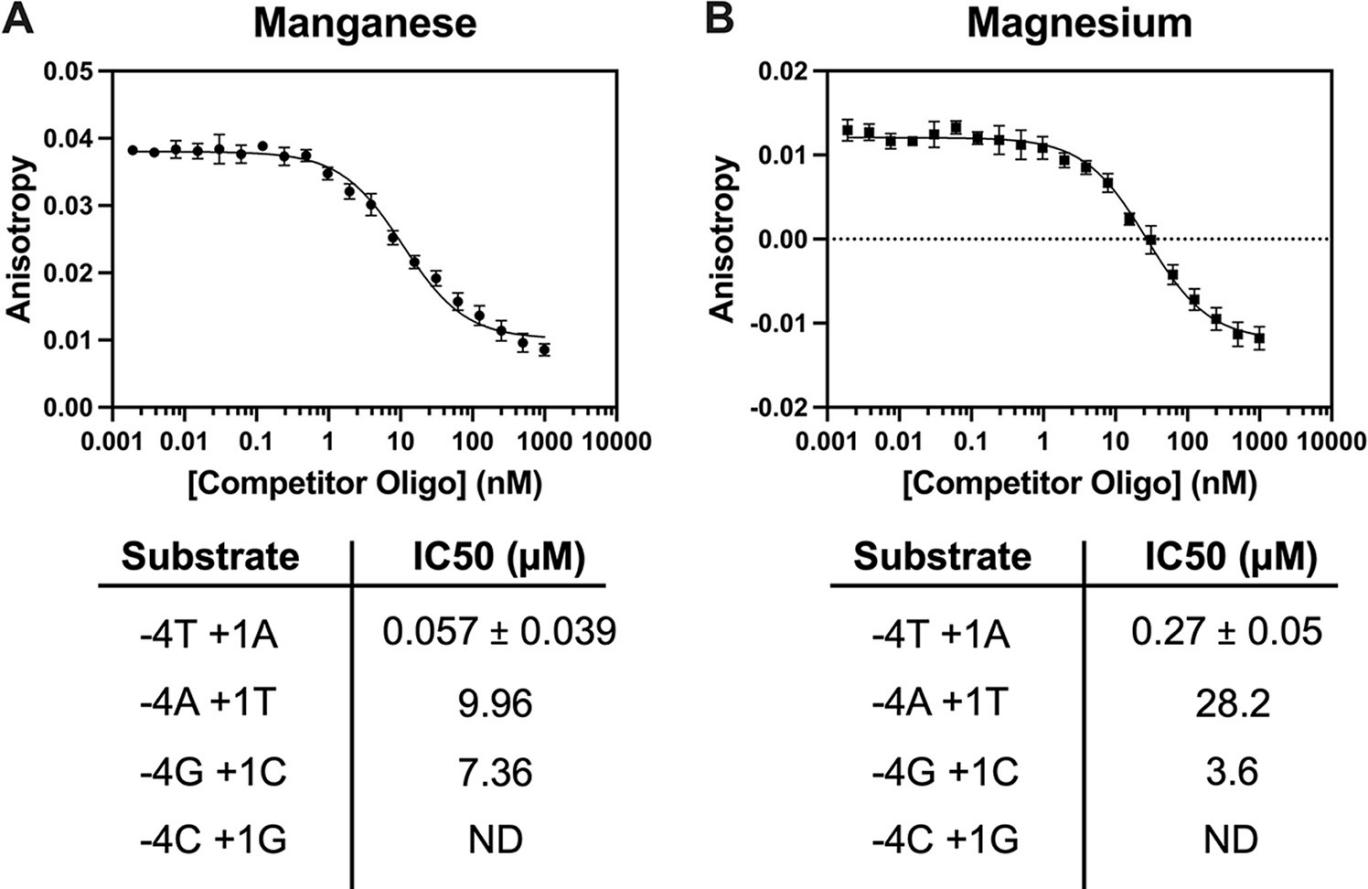
Sequence-dependent differences in dDCV Rep substrate binding. (A and B) Example fluorescence anisotropy competition binding curves with manganese (A) and magnesium (B) using unlabeled cognate competitor oligonucleotides above their respective tables of calculated IC_50_ values from mutagenic WC-containing competitor oligonucleotides. Each value was calculated from four independent replicates, with the WT substrate used as an internal control (*n* = 12).

We next aimed to determine the importance of the WC base pair between the −4T and +1 A in DNA binding. The sequence specificity of DCV Rep cleavage was recently profiled extensively using a next-generation sequencing (NGS) approach, HUH-seq, which extensively characterized preferred nucleotides across most of the Ori sequence (6). However, one limitation of our previous study is that, due to library size constraints, only the bases 5′ of the cleavage site were profiled, masking variations in the −4 and +1 bases comprising the WC base pair. Thus, we performed competition assays using fluorescence polarization to dissect the importance of the substrate’s WC base pair on Rep-ssDNA binding. We calculated the concentration of competitor at which half of maximal inhibition is achieved (IC_50_) values of unlabeled mutagenized competitor ssDNA oligonucleotides in competition with the fluorescent cognate sequence (Fig. 4A and B).

We first calculated IC_50_ values in the presence of Mn^2+^ and Mg^2+^ for the cognate sequence at 0.057 and 0.27 μM, respectively (Fig. 4A and B; Table S3); these values are roughly 10-fold higher than *K_D_* values under identical conditions. To validate our assay, we tested single base mutations of the cognate sequence and compared them to results of our previous studies using HUH-seq to assess sequence preferences of viral reps. The HUH-seq data showed that positions −7, −6, −5, −3, and −2 display no obvious base preference in the sequence logo for DCV, while bases at −4 and −1 are highly enriched for thymine. Detectable binding curves were measured for mutations of positions −7, −6, −5, −3, −2, and +3, whereas no quantifiable curves could be produced upon mutation of positions −4, −1, +1, and +2, in line with our previous results (Table S3). The undetectable competition results—meaning the sequences that were unable to compete off the cognate Ori—are also congruent with the previously determined cleavage specificity, which indicates a massive preference for thymine bases in the −4 and −1 positions. Additionally, these negative results also highlight the importance of the +1 A and +2 C bases, which were undetectable by the HUH-seq assay. However, our structural data also hint at the impact of the +2 cytosine in DCV Rep-substrate interaction, as this base makes a considerable number of contacts with the nuclease and is buried deep into its surface (Fig. S2B). Furthermore, our described structures suggest the importance of the substrate’s +1 position as well because it forms the previously mentioned stabilizing WC base pair, a feature present in all ssDNA-bound Rep structures solved thus far. We hypothesize that this intramolecular substrate pairing stabilizes its U shape and promotes binding and function more broadly.

10.1128/mbio.02587-22.5 TABLE S2 Table of crystallographic data collection and refinement statistics. Download Table S2, DOCX file, 0.01 MB. Copyright © 2022 Smiley et al. 2022 Smiley et al. https://creativecommons.org/licenses/by/4.0/ This content is distributed under the terms of the Creative Commons Attribution 4.0 International license.

10.1128/mbio.02587-22.6 TABLE S3 IC_50_ values measured via fluorescence polarization competition assays using mutagenized competitor oligonucleotides against a labeled oligonucleotide derived from the DCV Rep cognate Ori sequence. (Top) IC_50_ values of competitor oligonucleotides harboring a point mutation. Saturation mutagenesis was performed at positions −5 and +2 in the Ori sequence because of their importance in specificity and structure, respectively. Further single-transition mutations were characterized at all other positions across the Ori sequence. (Bottom) IC_50_ values for each of the 16 potential base combinations between the −4 and +1 positions, which form a proper WC base pair in all elucidated crystal structures of Reps in complex with ssDNA. The cognate sequence was used as an internal comparison for all competition experiments, hence the high number of replicates. Download Table S3, DOCX file, 0.01 MB. Copyright © 2022 Smiley et al. 2022 Smiley et al. https://creativecommons.org/licenses/by/4.0/ This content is distributed under the terms of the Creative Commons Attribution 4.0 International license.

We tested whether disruption of this WC base pair modulated affinity via further competition assays using a library of oligonucleotides containing substitutions of each of the four bases at both the −4 and +1 positions. Interestingly, we obtained measurable IC_50_ values only for the cognate sequence and the −4A·+1T and −4G·+1C double transversions (Fig. 4A and B). All other sequences in the library were unable to compete with the cognate substrate, including the size-similar −4C·+1G double transition. This suggests that noncognate WC-forming double substitutions are able to restore base pair formation, and thus binding, but perhaps with various degrees of success.

These results indicate the WC base pair is a key element to the initial interaction and binding of the DNA by viral Reps.

### The WC base pair is required for DNA cleavage.

**FIG 5:**
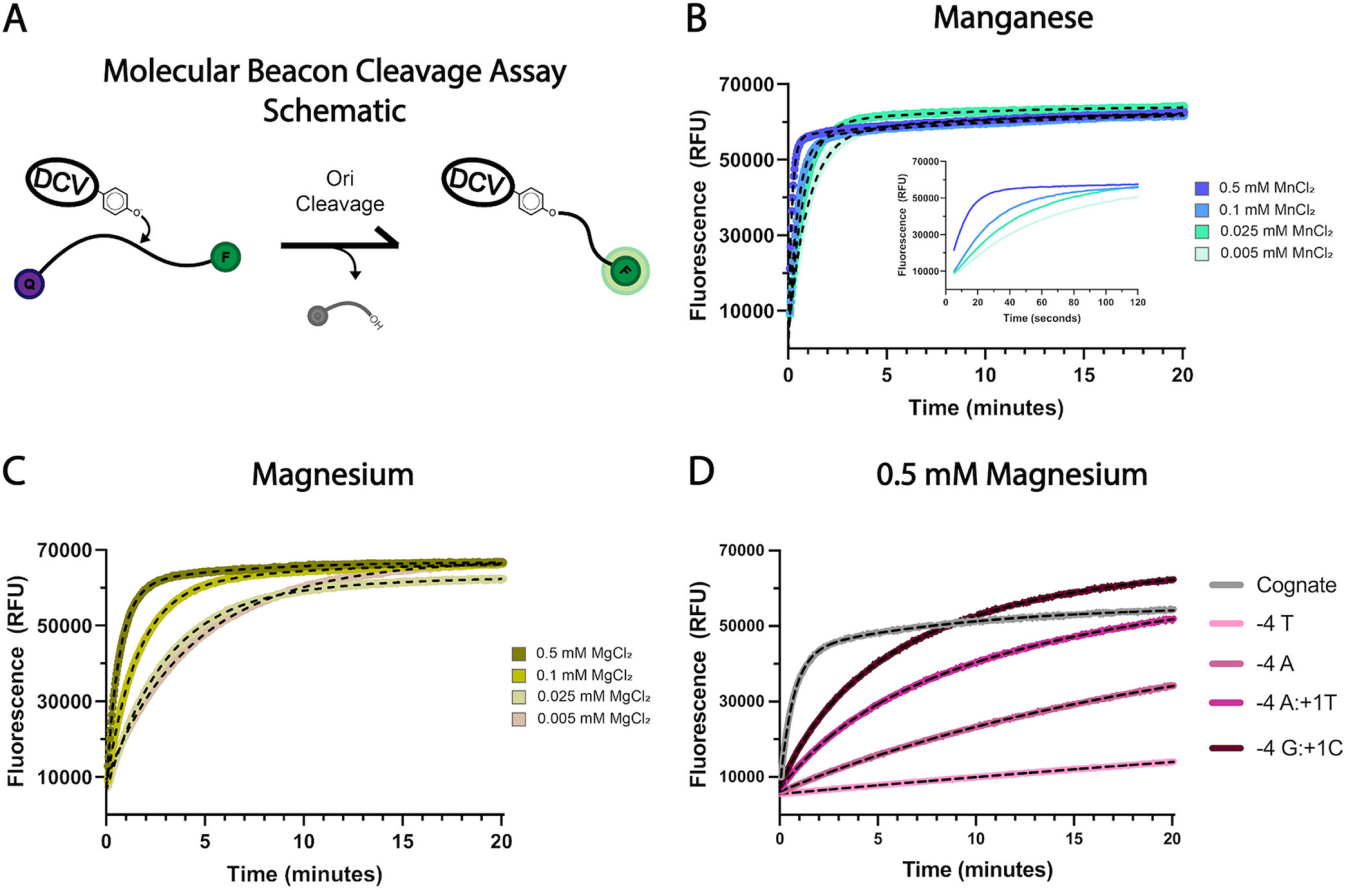
Molecular beacon cleavage reactions probing DCV Rep-mediated cleavage metal dependency and substrate sequence/structure requirements. (A) Graphical depiction of the molecular beacon cleavage assay in which DCV cleaves a dually labeled fluorophore-quencher oligonucleotide, physically separating the fluorophore from the quencher and remaining covalently bound to the fluorescent end of the substrate. (B and C) Molecular beacon cleavage reactions catalyzed by DCV on cognate Ori sequence-derived fluorophore-quencher oligonucleotides in the presence of various concentrations and identities of divalent cation. (D) Further molecular beacon cleavage reactions catalyzed by DCV on noncognate substrates containing mutations to the WC-forming bases in the presence of 0.5 mM magnesium.

Viral Reps are known to rapidly and robustly cleave their cognate sequences *in vitro*. We first sought to characterize DCV Rep cleavage of its cognate sequence by defining cleavage rates under a range of conditions to assess whether cofactor identity and concentration affect cleavage rate congruent with binding affinity data. Due to the high rate of cleavage, we employed a previously described (7) continuous molecular beacon plate reader-based assay where cleavage is read out by an increase in fluorescence upon cleavage of a synthetic dually labeled quencher-fluorophore oligonucleotide derived from the Ori sequence of DCV Rep (Fig. 5A). DCV Rep cleaved over 95% of its substrate in well under a minute in the presence of 0.5 mM Mn^2+^, with an average fast-rate constant (*k*_fast_) of 7.42 min^−1^, over four times faster than in the presence of Mg^2+^, which has a *k*_fast_ of 1.67 min^−1^ (Fig. 5B and C; Table 1). Similar to binding affinity trends, decreasing concentrations of Mg^2+^ and Mn^2+^, or increasing concentrations of NaCl, substantially lower the cleavage rate (Fig. 5B and C; Table S4).

**TABLE 1:** Cleavage reaction rates of DCV Rep mutant substrate reactions in the presence of 0.5 mM of either magnesium or manganese calculated by fitting the gain of fluorescence molecular beacon cleavage data to a one- or two-phase decay^*a*^

Sequence (5′→3′)	Name	Reaction rate with 0.5 mM of:			
		Mn^2+^		Mg^2+^	
		*k*_fast_ (min^−1^)	*k*_slow_ (min^−1^)	*k*_fast_ (min^−1^)	*k*_slow_ (min^−1^)
T A T T A T T * A C	D-*ori*	7.42 ± 1.66	0.09 ± 0.04	1.67 ± 0.24	0.22 ± 0.12
• • • A • • • * • •	−4A	0.047 ± 0.003		<0.01	
• • • • • • • * T •	+1T	0.010 ± 0.003		<0.01	
• • • A • • • * T •	−4A·+1T	8.18 ± 3.13	0.08 ± 0.02	0.32 ± 0.09	0.07 ± 0.02
• • • G • • • * C •	−4G·+1C	7.98 ± 0.80	0.09 ± 0.02	0.45 ± 0.03	0.12 ± 0.01

*^a^*The results are reported as the mean cleavage rate across three replicates, including standard deviation values.

10.1128/mbio.02587-22.7 TABLE S4 (Top) Cleavage reaction rates of DCV Rep cognate substrate reactions in the presence of a range of concentrations of either magnesium or manganese calculated by fitting the gain of fluorescence molecular beacon cleavage data to a one- or two-phase decay. (Bottom) Cleavage reaction rates of DCV Rep cognate substrate reactions in the presence of a range of 0.5 mM of either magnesium or manganese and a range of NaCl concentrations calculated by fitting the gain of fluorescence molecular beacon cleavage data to a one- or two-phase decay. Download Table S4, DOCX file, 0.01 MB. Copyright © 2022 Smiley et al. 2022 Smiley et al. https://creativecommons.org/licenses/by/4.0/ This content is distributed under the terms of the Creative Commons Attribution 4.0 International license.

Next, we assessed how mutations across the cognate substrate, including mutations that prevent WC base pairing, impact cleavage efficiency. Substrates that ablate WC base pair (T-4A and A·+1T) formation dramatically reduce cleavage efficiency to nearly undetectable levels, particularly in the presence of the less potent Mg^2+^ (Fig. 5D). However, double transversion mutations to the noncognate −4A·+1T and −4G·+1C WC base pairings restore cleavage efficiency to wild-type (WT) levels in reaction mixtures containing Mn^2+^ and are only modestly lower in reaction mixtures containing Mg^2+^ (Table 1). To further determine whether this stark decrease in catalytic efficiency upon mutation of the WC forming bases is the result of a perturbation in substrate conformation as opposed to a simple distortion of the specific sequence, we mutated the WC-adjacent −5T base to each of the other three possibilities and determined their impacts on cleavage efficiency by DCV Rep. Importantly, there is considerable variability in this base across the Ori sequences of different CRESS-DNA viral families (6). Surprisingly, none of the three mutant substrates were able to substantially reduce reactivity in the presence of Mn^2^, and efficiency was only mildly decreased in the presence of Mg^2+^ (Table S5), further corroborating our previous results that demonstrated huge plasticity in sequence specificity of viral Reps and further suggesting that reaction specificity is likely chiefly imparted by WC base pair formation.

10.1128/mbio.02587-22.8 TABLE S5 Cleavage reaction rates of DCV Rep −5 position mutant substrate reactions in the presence of 0.5 mM of either magnesium or manganese calculated by fitting the gain of fluorescence molecular beacon cleavage data to a one- or two-phase decay. Download Table S5, DOCX file, 0.01 MB. Copyright © 2022 Smiley et al. 2022 Smiley et al. https://creativecommons.org/licenses/by/4.0/ This content is distributed under the terms of the Creative Commons Attribution 4.0 International license.

### Noncognate WC base pairs result in highly efficient DNA processing.

**FIG 6:**
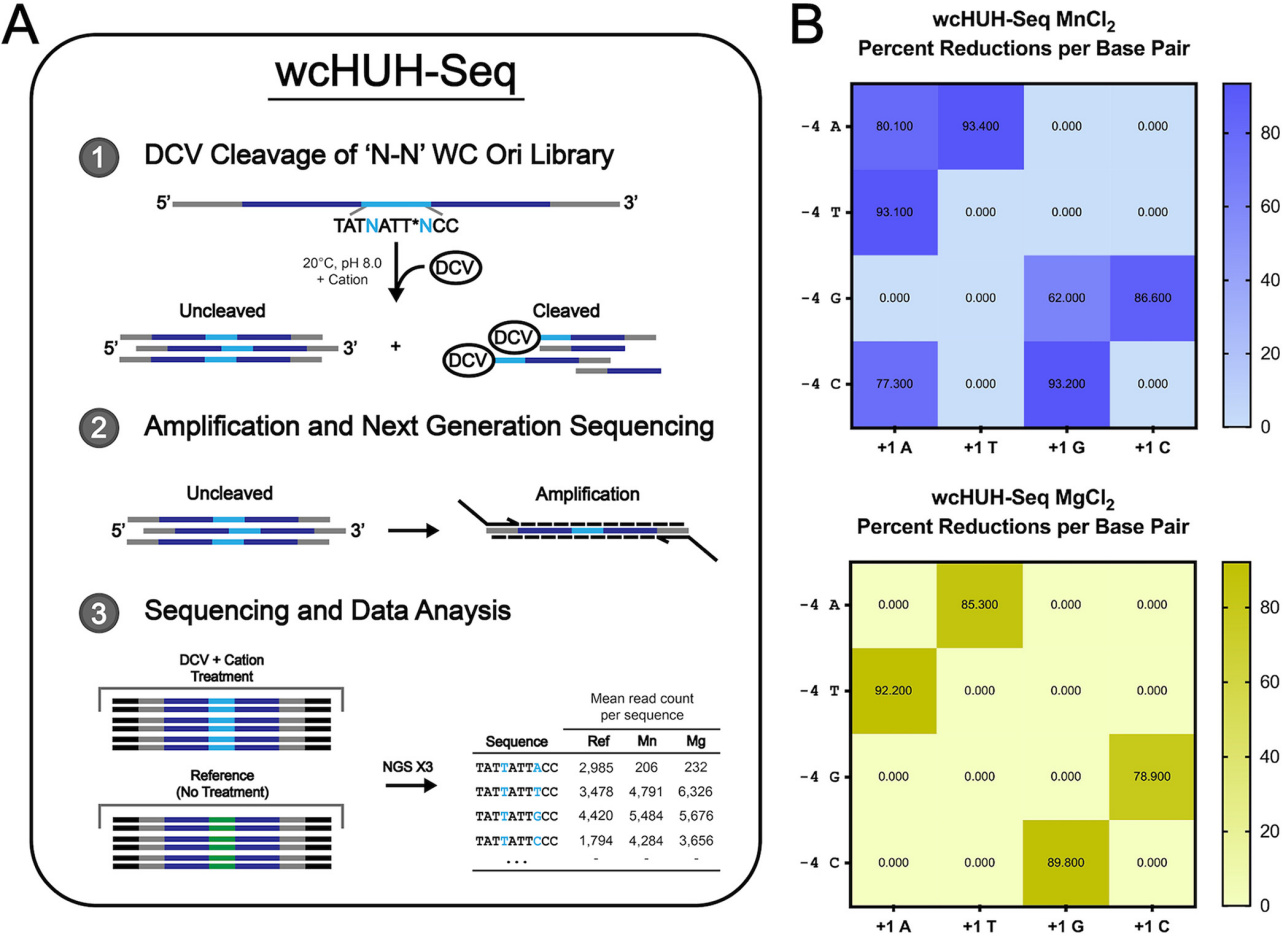
wcHUH-seq cleavage assay for determining the extent to which DCV Rep is able to act on the available range of canonical and noncanonical base pairings in the −4 and +1 positions of a cognate-derived substrate. (A) Schematic describing wcHUH-seq, an NGS-based approach to characterize the importance of intramolecular substrate WC base pairing in DCV Rep cleavage function. A synthetic oligonucleotide composed of a library derived from the cognate Ori sequence of DCV with the −4 and +1 positions randomized flanked by constant noninteracting regions and far 5′ and 3′ primer binding sites is reacted with DCV Rep in the presence of either manganese or magnesium. Uncleaved products are able to undergo PCR amplification using primers containing NGS sequencing adapters. Reacted amplified libraries and unreacted reference libraries are sequenced and used to determine percent reductions for each unique sequence via a custom Python-based analysis. Each sequencing reaction, including reference, was performed in triplicate across unique cleavage reactions. (B) Heatmaps indicative of the mean percent reduction in comparison to reference of each of the 16 available base combinations within an otherwise cognate DCV Ori sequence in the presence of 1 mM concentrations of either manganese (top) or magnesium (bottom).

Results from the described crystal structures and both the binding and functional assays reiterate the importance of substrate intramolecular WC base pairing in DCV Rep cleavage and binding, leading us to wonder to what extent, if at all, DCV Rep is able to act on substrates composed of each of the 16 base combinations in the WC-forming positions. Thus, we developed an NGS-based functional assay, Watson-Crick HUH-seq (wcHUH-seq), to examine the ability of DCV rep to cleave a cognate Ori-derived library of ssDNA containing all 16 base combinations in the base pair-forming −4 and +1 positions (Fig. 6A).

DCV was reacted under standard *in vitro* conditions in the presence of either manganese or magnesium using a cleavage library modeled after its cognate Ori sequence with the base pair forming −4 and +1 positions randomized (TAT**N**ATT*NCC). Reactions yield two populations of the library, cleaved and uncleaved sequences. Diluted cleavage reactions were used as a template for a PCR amplification with primers containing NGS sequencing adapters to amplify the uncleaved population exclusively, as the primer binding sites (PBS) become physically separated upon cleavage. The mean read counts from unreacted reference library replicates were used via substractive sequencing to calculate percent reductions for each of the 16 unique base combinations in manganese- and magnesium-treated cleavage reaction replicates (mean reference reads − mean experimental reads/mean reference reads). Heatmaps indicative of cleavage efficiency were generated using percent reductions for each unique sequence in each divalent metal reaction library with respect to the reference (Fig. 6A).

Strikingly, we are unable to detect any cleavage of non-WC base pair-forming substrates in the presence of MgCl_2_ with our wcHUH-seq assay, though both the canonical and noncanonical base pairs had substantial percent reductions in comparison to reference (Fig. 6B). Unsurprisingly, DCV Rep seems to have a minor preference for its cognate WC in comparison to any of the three other noncognate WC base pairings in the Mg^2+^ condition. However, the size-similar −4C·+1G pairing is cleaved at comparable levels to the cognate pairing, even though this variant was unable to demonstrate any discernible binding in the fluorescence polarization competition assays (Fig. 4A and B). Moreover, in the presence of MnCl_2_, substrates containing WC base pairs are still preferentially cleaved, all of them at surprisingly similar levels to the cognate substrate, but this intramolecular interaction is apparently no longer a prerequisite for cleavage in a narrow range of base combinations. More specifically, it seems that the presence of a +1 purine nucleotide in Mn^2+^ reactions limits the necessity of WC base pairing for substrate cleavage.

### WC base pair requirements in substrate reunion.

**FIG 7:**
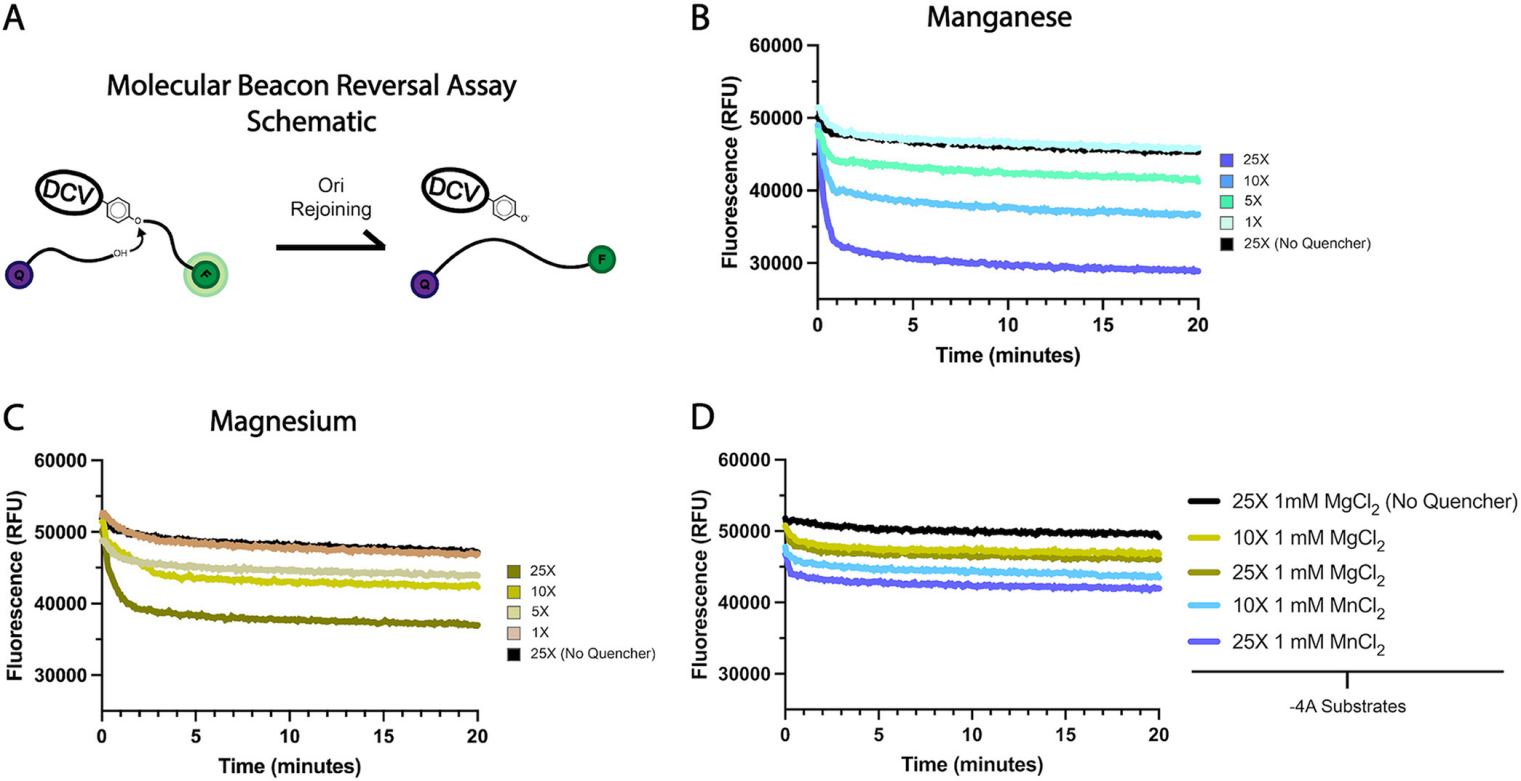
Molecular beacon reversal reactions probing DCV Rep-mediated substrate reunion metal dependency and sequence/structure requirements. (A) Graphical depiction of the molecular beacon reversal assay in which DCV rejoins a covalently linked fluorophore-labeled oligonucleotide to a quencher oligonucleotide, both derived from either the 5′ or 3′ side of the cognate substrate post cleavage. (B) Reversal assay with various mole ratios of reunion substrate to prelinked Rep Ori conjugates in the presence of 1 mM MnCl_2_. (C) Reversal assay with various mole ratios of reunion substrate to prelinked Rep Ori conjugates in the presence of 1 mM MgCl_2_. (D) Reversal assay with various mole ratios of mutant −4A reunion substrate to prelinked Rep Ori conjugates in the presence of 1-mM concentrations of either MnCl_2_ or MgCl_2_.

The final step of Rep-mediated RCR is the release of the phosphotyrosine covalent linkage between the Rep and the viral genome (or bacterial plasmid) via Rep-mediated intramolecular reunion of the cleavage-exposed 3′ end to the tyrosine-linked 5′ end for recircularization (Fig. 1A) (1). Though DCV and similar enzymes are known to release covalent linkages *in vitro* upon addition of a truncated version of its specific Ori sequence that excludes bases 3′ of the cleavage site (7, 26, 27), very little is known about the cofactor requirements, specificity, or efficiency of this interaction. Thus, we developed a continuous fluorescence-based intermolecular reversal reaction assay to characterize the efficiency of this reaction in the presence of different divalent cations and to determine if reunion, like cleavage, is dependent on substrate WC base pairing. The assay gauges substrate reunion over time through a loss of fluorescence readout via linkage of a quencher-labeled 3′-truncated Ori sequence and a DCV Rep-bound FAM-labeled substrate (Fig. 7A).

Interestingly, we again see that Mn^2+^ is a potent activator of DCV Rep function, as it yields a dramatically more substantial reduction in fluorescence than Mg^2+^ does, dropping signal by around 20,000 relative fluorescence intensities (RFUs) in comparison to the around 10,000 RFUs of Mg^2+^ with 1 mM concentrations of divalent and a 25× mole ratio of reversal substrate to DCV Rep-cleavage oligonucleotide covalent linkage (Fig. 7B and C). Furthermore, our results suggest that Mn^2+^ is better able to recruit substrate for ssDNA reunion, as its presence in only a 10× mole ratio of reversal substrate can elicit the same reduction in signal as Mg^2+^ with a 25× ratio. Surprisingly, DCV Rep-mediated substrate reunion seems to occur in the absence of divalent cations as demonstrated by the very modest reduction in signal in the presence of EDTA at higher mole ratios (Fig. S3). However, while this minimal reduction of signal suggests that the reunion reaction could be taking place in the absence of cation, further investigation is required to determine the roles that divalent ions play in substrate reunion. We also sought to characterize how preventing WC formation affects substrate reunion through further reunion experiments with a substrate containing a T-4A mutation that prevents −4·+1 substrate base pairing. In congruence with the results from both the cleavage and binding experiments, ablation of the WC base pair dramatically limits the ability of DCV Rep to perform its ssDNA reunion function regardless of the present cation (Fig. 7D), further demonstrating the near necessity of the WC base pair in Rep-ssDNA interaction, even on physically separated substrates.

10.1128/mbio.02587-22.3 FIG S3 Reversal assay with various mole ratios of reunion substrate to prelinked Rep Ori conjugates in the presence of 1 mM EDTA. Download FIG S3, TIF file, 2.4 MB. Copyright © 2022 Smiley et al. 2022 Smiley et al. https://creativecommons.org/licenses/by/4.0/ This content is distributed under the terms of the Creative Commons Attribution 4.0 International license.

**FIG 8:**
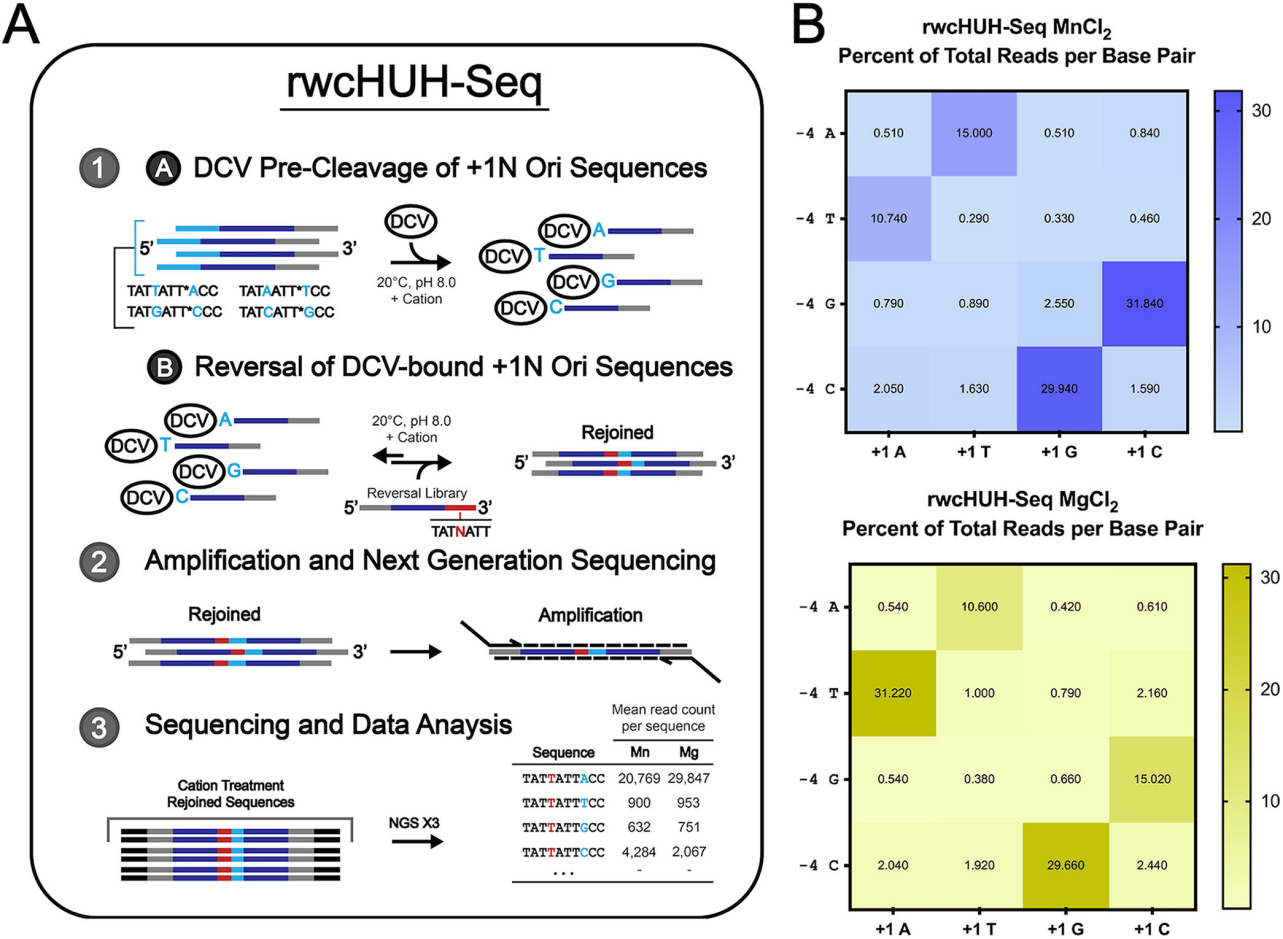
rwcHUH-seq cleavage assay for determining the extent to which DCV rep is able to rejoin substrate comprising the available range of canonical and noncanonical base pairings in the −4 and +1 positions of a cognate-derived pair of physically separated substrates. (A) Schematic describing rwcHUH-seq, an NGS-based approach to characterize the importance of intramolecular substrate WC base pairing in DCV Rep reversal function. DCV Rep is prereacted with a synthetic oligonucleotide library comprised of all available WC base pair combinations between the substrate’s −4 and + 1 positions such that DCV is covalently linked to each of the four different bases in the +1 position flanked by a constant noninteracting region and a 3′ primer binding site. Prelinked DCV Rep is then reacted with an oligonucleotide library derived from the sequence 5′ of the cleavage site with the −4 position randomized, a noninteracting constant region, and a far 5′ primer binding site. Reversed, and thus rejoined, oligonucleotide pairs then contain both 5′ and 3′ primer binding sites and can be specifically amplified via PCR, using primers containing NGS sequencing adapters, out of reaction pools. Each sequencing reaction is performed in triplicate and used to determine the mean percentage of total reads of each of the unique 16 bp comprising the canonical WC-forming positions via a custom python-based analysis. (B) Heatmaps indicative of mean percentage of total reads, and thus the magnitude of reversal, of each of the 16 available base combinations within an otherwise cognate pair of oligonucleotides comprising the DCV Ori sequence in the presence of 1 mM concentrations of either manganese (top) or magnesium (bottom).

To more extensively characterize WC requirements of DCV Rep-mediated substrate reunion, we developed a second NGS-based assay, reversal-wcHUH-seq (rwcHUH-seq). For rwcHUH-seq, DCV was prereacted with a 3′ PBS-containing Ori-derived sequence harboring one of the four possible base pairs (A·T, T·A, G·C, and C·G) such that the first nucleotide covalently linked to the nuclease post cleavage is either A, T, G, or C. The efficiency of linkage for each of these prereactions was determined in order to mix the four +1 nucleotide Ori-Rep conjugations in an equimolar concentration (Fig. S1). Following this, a 5′ PBS-containing cleaved Ori-derived reversal library containing a randomized −4 position (which canonically forms the WC base pair with the now covalently linked +1 position) was added to the solution containing the +1 nucleotide Ori-Rep conjugations in a 25:1 molar ratio in the presence of either MnCl_2_ or MgCl_2_. Incubated reversal reactions were diluted and used as a template for PCR amplification with primers containing NGS sequencing adapters in order to specifically amplify the rejoined sequences, as they are the only population that harbors both a forward and reverse primer binding site. Mean read counts across three replicates for each unique sequence were used to calculate the percentage that each of the 16 base combinations contributes to the total number of reads in the Mn^2+^ and Mg^2+^ reversal reaction conditions (Fig. 8A). Congruent with the wcHUH-seq assay, the reversal assay again demonstrates that reactions in the presence of MgCl_2_ are largely limited to substrates that form proper WC base pairings, but with a substantially larger preference for the cognate −4T·+1A and size-similar −4C·+1G pairings (Fig. 8B). In contrast with our cleavage assay, we see that reversal reactions in the presence of MnCl_2_ are less promiscuous, preventing substantial interactions with non-WC-forming substrates (Fig. 8B). However, reversal reactions in the presence of the more potent Mn^2+^ do seem more relaxed in terms of their preference for specific types or sizes of base pairings. Altogether, in congruence with our molecular beacon assays, the reverse reaction appears to be less dependent on divalent cations overall—certainly less sensitive to hyperactivation by manganese than the cleavage reaction—and perhaps somewhat more dependent on the substrate’s underlying sequence.

### Ori sequence WC base pair variability throughout CRESS-DNA viruses.

As we probed the necessity of WC base pairing in Rep-substrate interactions, we were surprised to see that though the cognate base pair was somewhat favored over others, its requirement for function was seemingly more structure specific than sequence specific. Considering the prevalence of WC base pairing in the substrates of all elucidated cocrystal structures of CRESS-DNA virus Reps thus far (6, 22), we speculated that this intramolecular interaction is a universal requirement for the cleavage and reversal functions of these proteins and that there are likely viral genomes that harbor concomitant mutations that yield Ori sequences containing other WC base pairs than the canonical −4T·+1A. Encouragingly, small subpopulations of geminivirus Ori sequences have already been reported to contain a +4G·−1C double transversion (28, 29). To characterize the extent to which noncanonical pairings occur in nature, we developed a Python script called Ori-Seek to search for putative Ori sequences across CRESS-DNA viral genomes. Inspired by the recent success of StemLoop-Finder (30), our script searches the available complete CRESS-DNA viral genomes downloaded from NCBI Virus (31) for a list of user-defined semiambiguous Ori sequences. Once an Ori has been located within a genome, the script then checks the surrounding sequence environment for stem-loop structures that meet a folding energetic cutoff and ultimately returns a list containing genome accession numbers and descriptions, putative Ori sequences, and stem-loop structures as predicted by ViennaRNA 2.0 (32).

Using a series of WC-ambiguous Ori sequence search terms derived from endogenous Oris (TAA**N**ATT**N**C, NAT**N**ATT**N**C, and NAG**N**ATT**N**C), we were able to predict putative Ori sequences for 83.8% of the genomes queried, or 21,566 predictions out of 25,735 searched genomes (Tables 2 and 3). Surprisingly, we found 104 noncognate swaps out of the total 13,537 total predictions using the geminivirus-like TAA**N**ATT**N**C Ori search term, with the vast majority of them (100 of the 104) corresponding to size-dissimilar −4G·+1C swaps, one −4A·+1T swap, and three size-similar +4C·−1G swaps (Tables 2 and 3 and Table S7). The abundance of −4G·+1C swaps corroborates our *in vitro* competition results that demonstrated this sequence was the best able to compete off the wild-type substrate (Fig. 4A and B). Searches using the circovirus-like NAT**N**ATT**N**C and nanovirus-like NAG**N**ATT**N**C search terms yielded 1,801 and 6,228 predictions, respectively (Tables 2 and 3 and Table S7). Of the circovirus-like predictions, only a single representative contains the putative swap of −4A·+1T. Finally, of the nanovirus-like predictions, five representative sequences contain this same −4A·+1T swap (Tables 2 and 3 and Table S7). Though size dissimilar, this −4A·+1T swap again corroborates our *in vitro* data that demonstrated that this swap was one of two noncognate WC pairings with detectable binding in competition experiments (Fig. 4A and B). Altogether, our findings demonstrate that concomitant mutations which ablate the canonical WC base pair sequence but preserve the structure via a swap to a different proper WC pairing do occur in nature, but in very low abundance.

**TABLE 2:** Total Ori-Seek predictions indicating the counts and percentage of total predictions as well as counts and percentages of cognate WC-containing, noncognate WC-containing, and WC-lacking predictions

Ori-Seek prediction	No. of counts	% of parent
Total genomes	25,735	
Total predictions	21,566	83.8
Total cognate WC	21,433	99.38
Total noncognate WC	110	0.51
Total non-WC	23	0.11

**TABLE 3:** Total Ori-Seek predictions grouped by the TAANATTNC, NATNATTNC, and NAGNATTNC semiambiguous Gemini-, Circo-, and Nano-like search terms, respectively

Search term	Gemini-like	Circo-like	Nano-like
Total predictions	13,537	1,801	6,228
−4T·+1A	13,420	1,794	6,219
−4A·+1T	1	1	5
−4C·+1G	3	0	0
−4G·+1C	100	0	0
Non-WC	13	6	4

10.1128/mbio.02587-22.10 TABLE S7 List of genome accession numbers and genome descriptions that have predicted Ori sequences that are putatively unable to form WC base pairs (left) in line with accession numbers and genome descriptions from different isolates of the same viral species with predicted Ori sequences that are putatively able to form WC base pairs (right). Download Table S7, DOCX file, 0.02 MB. Copyright © 2022 Smiley et al. 2022 Smiley et al. https://creativecommons.org/licenses/by/4.0/ This content is distributed under the terms of the Creative Commons Attribution 4.0 International license.

The strong corroboration of our *in vitro* and genomic data suggest that substrate WC base pairing in CRESS-DNA viral Ori sequences may be a universal requirement for interaction with Rep proteins. To further interrogate this possibility, we searched the remaining 4,169 genomes that our script could not form predictions for in search of putative Ori sequences that lack a WC base pairing. In total, this search provided an additional 23 putative Ori sequences, all lacking the ability to form a proper −4·+1 WC base pair (Table S6). Again, the geminivirus-like TAA**N**ATT**N**C search term resulted in a rough majority of predictions with 13 additional putative Oris, and the circovirus-like NAT**N**ATT**N**C and nanovirus-like NAG**N**ATT**N**C search terms yielded 6 and 4 additional predictions, respectively (Table S6). Overall, there is no obvious skew toward mutation of the −4 or +1 positions in these predicted Ori sequences, nor is there an overt preference for sequence content (i.e., AT or GC bias) in the mutations. Furthermore, a number of these predicted Oris are from viral genomes that have been predicted to have WC pairings in other isolated genomes by our Ori-Seek script (Table S7). The low abundance of these WC-lacking Oris paired with this prediction discrepancy suggests that some of these predictions are the result of erroneous amplification or sequencing. However, it does appear that a minority of these WC-lacking predictions are genuine and have been previously reported in the literature, such as the AATAATT*AC Ori from the chimpanzee-associated porpismacovirus (GenBank accession no. NC_039068) (33).

10.1128/mbio.02587-22.9 TABLE S6 (Top) Counts of each unique WC-containing prediction by Ori-Seek grouped by search term. Sequences in blue are canonical Ori sequences for the search term. Sequences in black are either sequences that are less widespread or WC swaps. Base-pairing positions −4 and +1 are bold and underlined. (Bottom) Counts of each unique WC-lacking prediction by Ori-Seek grouped by search term. Base-pairing positions −4 and +1 and bold and underlined. Download Table S6, DOCX file, 0.01 MB. Copyright © 2022 Smiley et al. 2022 Smiley et al. https://creativecommons.org/licenses/by/4.0/ This content is distributed under the terms of the Creative Commons Attribution 4.0 International license.

## DISCUSSION

The reported cocrystal structures of DCV Rep in complex with its cognate substrate, with and without a coordinated manganese, add an interesting ion-focused perspective to the expanding repertoire of structures that describe the molecular basis of Rep-ssDNA interaction (6, 22, 34). A common feature across all structures of CRESS-DNA viral Reps in complex with their ssDNA substrates elucidated thus far is an intramolecular WC base pair between the −4T and +1A positions with respect to the cleavage site. We show that this substrate interaction is conserved in the absence of a coordinated cation, suggesting that the Rep facilitates this pairing independently of the polarization and positioning imparted by the catalytically required metal. This structural comparison, along with further binding experiments, demonstrates that viral Reps are able to bind their substrates in the absence of cation, though with considerably lower affinity and different positioning. Altogether, this suggests that substrate coordination and priming for cleavage is a concerted effort between the enzyme, which binds the substrate and facilitates base pairing, and the cation, which both polarizes and pulls the ssDNA backbone into a position conducive to cleavage.

The prevalence and ion independence of the conserved intramolecular substrate WC base pairing highlighted its potential importance in Rep-ssDNA interaction. While this substrate feature had been previously identified in the small number of resolved cocrystal structures of Reps in complex with their substrates, it was unknown to what extent it was involved in Rep-substrate interaction and unclear how conserved it was across the rapidly expanding CRESS-DNA viral family. In this work, we demonstrated that this pairing is a critical component of Rep-substrate binding, cleavage, and reunion through fluorescence-based functional assays (Fig. 4, 5, and 7). Remarkably, we showed that noncognate WC base pair swaps are generally well tolerated in DCV Rep-substrate interaction, suggesting that specificity in Reps is driven by both the substrate’s underlying sequence and its secondary structure. This structural preference suggested that Ori sequences harboring seemingly noncognate swaps may exist in nature. Thus, we developed the Ori-Seek script, which searches the genomes of CRESS-DNA viruses for semiambiguous Ori sequences and examines the surrounding sequence environment for Ori-like hairpin formations. Ori-Seek was able to make predictions for just under 84% of the 25,735 genomes queried, with 99.89% of the total predictions containing putative WC base pairs between the −4 and +1 positions and 0.11% unable to pair between these positions (Tables 2 and 3). Of the WC-containing putative Oris, 0.51% of them harbor noncognate WC base pair swaps to one of the three other pairings, with a major preference for the −4G·+1C mutant. The overwhelming majority of predicted Oris are putatively able to form WC base pairs between the −4 and +1 positions with respect to the cleavage site. The widespread presence of this potential intramolecular interaction paired with our *in vitro* data suggest that WC base pairing is a universal requirement for Rep-ssDNA interaction in CRESS-DNA viruses.

Interestingly, recent structural analyses and computational modeling of the HUH-containing endonuclease domain from the distantly related parvovirus B19 NS1 protein (B19) suggest that this intramolecular substrate base pairing may be broadly conserved across replication-initiating HUH endonucleases (35). However, though the model of B19-ssDNA interaction predicts the presence of a −4G·+1C base pairing, previous investigations into the sequence specificity of this enzyme indicate that it may not be functionally dependent on this pairing (36). Unintuitively, ablation of the putative B19 substrate WC base pairing via −4G to C markedly increases cleavage efficiency, whereas the +1C to G mutation nearly inhibits cleavage entirely. Though this conflicting data does not necessarily preclude a conservation of substrate architecture, including WC base pairing, between these distantly related enzymes, it does indicate that they may have different substrate conformation requirements for nucleolytic action.

Looking beyond replication-initiating HUH endonucleases to the relaxase subfamily, we see greater variety and complexity in the conformations and underlying sequences of substrates across its representatives. TrwC, the MOB_F_ relaxase of plasmid R388, interacts with its nucleic acid target, OriT, through a network of inter- and intramolecular interactions between the protein and its substrate that are considerably more complex than the Rep-ssDNA interaction (37–39). This complexity in substrate recognition is maintained across other MOB_F_ relaxase representatives, like TraI of plasmid F (23, 40, 41). Similar to Reps, both of these relaxases conform their substrates into distinct U shapes, as noted previously (6). However, these shapes are not mediated by WC base pairing but, rather, by a mix of intramolecular hydrogen bonding and stacking between bases in the substrate. This difference in substrate self-interaction, paired with requirements of distal hairpin binding in relaxases, indicates that, though the substrate architectures of these related enzymes are superficially similar to that of Reps, the means by which they achieve this conformation and how these interactions regulate cleavage are quite different.

In comparison to the cleavage and covalent linkage functions of Reps, the reunion of substrates by these enzymes is comparatively understudied. To our knowledge, outside a small number of instances that demonstrate this function and a minimal investigation into its sequence specificity, this reaction remains poorly understood (7, 26, 27). This study provides additional mechanistic insight into the rejoining reaction catalyzed by Rep proteins post-substrate cleavage. Specifically, we demonstrate that though the extent of substrate reunion is dramatically enhanced by divalent cations, the reaction is not catalytically dependent on them, as demonstrated by the modest reduction of fluorescent signal in our molecular beacon reversal assays (Fig. 7; see Fig. S3 in the supplemental material). This cation independence is in stark contrast to the well-known requirements of coordinated divalent metals for cleavage and covalent linkage in HUH endonucleases (1). Moreover, we demonstrate that rejoining, along with binding and cleavage, of ssDNA substrates is dependent on the structurally conserved intramolecular substrate WC base pairing between the −4 and +1 positions. The functional requirement of this intramolecular substrate interaction leads us to speculate that the physically separated substrate (i.e., everything 5′ of the cleavage site) returns to the active site in a precleavage-like conformation, which promotes intersubstrate base pairing and brings the freed 3′ OH into proximity of the phosphotyrosine for subsequent attack and ligation.

The uncovered functional dependence of substrate WC base pairing adds additional utility to these enzymes in their applications as fusion partners, or HUH-tags, for sequence-directed protein-ssDNA bioconjugation. As previously mentioned, our prior attempts to define the sequence specificity of these enzymes excluded the positions 3′ of the cleavage site, which prevented interrogation of the critical +1A base. This additional base dramatically expands the sequence space available to search for intrinsically orthogonal HUH-tags, which could allow for more opportunities to multiplex these nucleases without the need for protein engineering. Expanding the capacity of Reps for multiplexing would further their applicability in DNA-based technologies and techniques, like DNA-PAINT superresolution microscopy (42), sequencing-based epitope quantification (43), and proximity ligation (44). Moreover, the reverse reaction could have potential applications in user-defined temporal control of DNA-based utilities like cell surface labeling with fluorescent oligonucleotides (45) or DNA-mediated liposome fusion in synthetic cells (46) by providing a simple and efficient means of releasing Rep-ssDNA covalent linkages.

We believe that our *in vitro* data paired with the overwhelming prevalence of putative WC base pairings across CRESS-DNA viral genomes makes a strong case for substrate intramolecular WC base pairing as a universal requirement of ssDNA cleavage and reunion in Rep HUH endonucleases. Our wcHUH-seq method provides strong *in vitro* support for this claim, particularly in magnesium conditions, which show no substrate cleavage in the absence of a −4·+1 WC base pairing. However, reactions in the presence of manganese somewhat stray from our conclusions in that the DCV rep is able to cleave substrates with −4A·+1A, −4C·+1A, and −4G·+1G noncannonical base pairings. Manganese is known to hyperactivate nucleases and polymerases, which may be what is enabling this Rep to act on these otherwise inaccessible substrates. This discrepancy in activity, paired with the cellular abundance of magnesium and toxicity of high concentrations of manganese, suggests that the endogenous metal ion required for substrate cleavage by Reps is likely to be magnesium. Our genomic analysis, Ori-Seek, was designed to predict the Ori sequences of all complete CRESS-DNA viral genomes available on NCBI Virus (31) at the time of the preparation of the manuscript. Importantly, these predictions are based on an initial search for an ambiguous variant of a known Ori sequence and a subsequent search that determines if the identified sequence is at least partially incorporated into the loop of a stem-loop comprised of its surrounding sequence as predicted by ViennaRNA 2.0 (32). Thus, this analysis is only able to predict Ori sequences that are both relatively similar to those previously identified and able to form stem-loop structures with their surrounding sequence. While we were able to predict Oris using these restraints for a majority of available genomes (83.8%), the remaining 16.2% likely have sequences that vary from these restraints in some way. These unpredicted genomes could harbor Ori sequences that differ from those that are well established in any number of ways, preventing certainty of a universal requirement of intra-Ori WC base pairing. Considering the ever-expanding diversity of the CRESS-DNA virosphere (47), we expect our understanding of their Ori sequences to improve over time, further illuminating their conserved sequence content and secondary structure.

Altogether, these findings build upon our current understanding of Rep-ssDNA recognition. A more comprehensive characterization of the minimal requirements for substrate cleavage and covalent linkage by Reps will further drive their development as functional fusion proteins for ssDNA labeling applications. Additionally, this work deepens our knowledge of Rep-mediated substrate reunion, demonstrating it as a rapid and robust means by which to release Rep-DNA bioconjugations, which could serve to further expand the utility of HUH-tags.

## MATERIALS AND METHODS

### Molecular cloning.

The 6×His-SUMO-DCV Rep plasmids were generated in a previous study (6). Briefly, the DCV Rep sequence (amino acids 1 to 105; GenPept accession no. AAR28041) was cloned into the pTD68/6×His-SUMO expression vector as a gene block (IDT) with the In-Fusion HD cloning kit (TaKaRa) using BamHI and XhoI restriction sites (NEB). The inactive dDCV point mutant was created via site-directed mutagenesis using the same cloning kit per the manufacturer’s instructions and the SUMO-DCV vector as a template. 6×His-SUMO-MBP-DCV constructs were cloned by inserting the maltose binding protein (MBP) gene on the N terminus of the DCV Rep sequence. Each plasmid was sequence confirmed with Sanger sequencing (Genewiz).

### Protein construct choice, expression, and purification.

Three separate Rep constructs were used throughout the manuscript across a variety of assays. Briefly, untagged DCV Rep was used in all fluorescence- and NGS-based cleavage and reversal assays as well as in X-ray crystallography. N-terminal SUMO-DCV Rep fusions were used in SDS-PAGE analysis of Rep-ssDNA cleavage and covalent linkage because the increase in molecular weight of the fusion protein enhances clarity in these assays. Finally, N-terminal MBP-DCV Rep fusions were used in fluorescence anisotropy assays to increase the change in anisotropy signal through greater molecular weight and thus slower rotation in solution.

Rep constructs were expressed in BL21(DE3)-competent E. coli cells (Agilent) in a 1-L volume with LB broth. The temperature was reduced from 37°C to 18°C after the optical density at 600 nm (OD_600_) reached 0.6, and cells were induced with 0.5 mM isopropyl-β-d-thiogalactopyranoside (IPTG; Sigma-Aldrich) and grown overnight. Cells were lysed in lysis buffer (50 mM Tris, pH 7.5, 250 mM NaCl, and 1 mM EDTA) with a complete protease inhibitor tablet (Pierce) and pulse sonicated at 4°C. The clarified supernatant was batch bound with Ni-nitrilotriacetic acid (NTA) HisPur agarose beads (Thermo Fisher) for 1 h, loaded onto a gravity column, washed with 30 column volumes of wash buffer (50 mM Tris, pH 7.5, 250 mM NaCl, 1 mM EDTA, and 20 mM imidazole), and finally eluted with elution buffer (50 mM Tris, pH 7.5, 250 mM NaCl, 1 mM EDTA, and 250 mM imidazole). Approximately 30 μg 6×His-ULP1 (SUMO-specific protease) per L of culture was added to the protein samples and dialyzed (50 mM Tris, pH 7.5, 250 mM NaCl, 1 mM EDTA, and 1 mM dithiothreitol [DTT]) overnight at 4°C. Protein samples were batch bound a second time with Ni-NTA HisPur agarose beads to remove cleaved 6×His-SUMO and 6×His-ULP1. Protein was further purified and buffer exchanged using the Superdex 300 increase 10/300 GL (GE Healthcare) size exclusion column into 50 mM Tris, pH 7.5, 300 mM NaCl, and 1 mM EDTA for storage. For crystallography, dDCV was concentrated to 35 mg/mL using Amicon Ultra-15 centrifugal spin concentrator (3-kDa cutoff filter; Sigma-Aldrich), and for biophysical experiments, DCV-MBP and MBP-DCV constructs were concentrated to 100 to 300 μM (10-kDa cutoff filter).

### Crystallization, data collection, and processing.

dDCV with 10-mer crystal hits were obtained using a broad screen provided by Rigaku’s CrystalMation sitting drop system. The dDCV solution contained 20 mg/mL protein, 1.8 mM 10-mer oligonucleotide (5′-dTATTATTACC-3′), and 7.5 mM MnCl_2_. Follow-up manual screening using 1:1 protein solution to well solution produced large orthorhombic crystals (300 by 100 μm on average). The well solution contained 0.1 M sodium acetate, pH 4.8, 20 mM CaCl_2_, and 26% 2-methyl-2,4-pentanediol. Crystals were soaked in 25% glycerol prior to snap freezing with liquid nitrogen. A 1.30-Å resolution data set was collected at the APS Beamline 24 (NE-CAT). The crystal belongs to the I 2 2 2 space group with unit cell parameters *a *= 55.4.17, *b *= 66.343 Å, and *c *= 71.259 Å, with 1 protein-DNA complex per asymmetric unit. Medium-sized hexagonal bipyramid-shaped crystals (100 by 75 μm on average) were obtained using the same dDCV protein plus DNA sample using manual screening with a well solution containing 0.1 M sodium acetate, pH 4.6, and 2.2 M ammonium sulfate. Crystals were soaked in 25% glycerol prior to snap freezing with liquid nitrogen. A 1.69-Å resolution data set was collected at the APS Beamline 24. The crystal belongs to the P 6_1_ 2 2 space group with unit-cell parameters *a *=* b *= 102.99 Å, *c *= 138.29 Å, and 2 protein-DNA complexes per asymmetric unit. All data were processed using the HKL suite.

### Structure solution and refinement.

Structures from both the I 2 2 2 and P 6_1_ 2 2 space groups were solved with the Phenix Phaser molecular replacement function using the structure of PCV Rep bound to a 10-mer (PDB accession no. 6WDZ) as the homology model. Coot was used for visual reconstruction alternated with refinement using PHENIX.auto.refine. Manganese was found to be bound in the active site of the 1.30-Å resolution structure and has an *R*_work_ and *R*_free_ of 0.149 and 0.186, respectively. Structure factors and coordinates were deposited into PDB under accession no. 7KII. Electron density for an ion in the active site of either subunit in the 1.69-Å resolution structure is not supported. Instead, density-supporting four waters were found in the active site spaces. The *R*_work_ and *R*_free_ statistics are 0.153 and 0.178, respectively, and structure factors and coordinates of this structure were deposited into PDB under accession no. 7KIJ.

### Simulated annealed omit maps.

Simulated annealed omit maps (m*F*_o_-D*F*_c_) to assess the presence of Mn^2+^ and water in the active site were generated using the Phenix composite omit map function using final coordinates and structure factors of the two dDCV plus 10-mer structures with all solvent molecules removed from the PDB file prior to map generation. Omit maps are visualized and contoured (σ = 2 or 3) using PyMol (Fig. S2C).

### Analytical size exclusion chromatography.

A Superdex 300 increase 10/300 GL size exclusion column was calibrated using gel filtration standards (Bio-Rad) according to the manufacturer's recommendations using an NGC liquid chromatography system (Bio-Rad). Next, 100 μL of 13 mg/mL purified recombinant MBP-dDCV was loaded onto the column calibrated in (50 mM Tris, pH 8.0, 150 mM NaCl, 1 mM DTT), and 1 mM EDTA or 5 mM MgCl_2_. The size of MBP-dDCV in solution was approximated using the standardized chromatogram (*A*_280_) normalized to 100 based on the highest peak and plotted using GraphPad Prism.

### *In vitro* HUH reactions.

*In vitro* oligonucleotide cleavage reactions were carried out using final concentrations of 3 μM SUMO-DCV and 30 μM oligonucleotide in a buffer composed of 50 mM HEPES, pH 8.0, 50 mM NaCl, 1 mM DTT, and 1 mM divalent cation (MnCl_2_ or MgCl_2_). Reaction mixtures were incubated at room temperature for 30 min before subsequent denaturation and SDS-PAGE analysis.

### Fluorescence anisotropy assays.

The DCV Rep’s affinity for its ssDNA-FAM substrate (IDT) was measured by titration of MBP-dDCV via 1:2 serial dilution starting at 1 μM into a 10-nM solution of the labeled substrate in cleavage reaction buffer composed of 50 mM HEPES, pH 8.0, 1 mg/mL bovine serum albumin (BSA), 0.05% Tween 20, and 1 mM DTT, with variable NaCl and divalent cation concentrations depending on the experiment. Four separate reaction mixtures were assembled in quadruplicate, each using four rows across a 384-well plate. Reaction mixtures were incubated for 30 min at room temperature to ensure equilibrium and centrifuged at 2,000 × *g* for 2 min before measurement. Dissociation constants (*K_D_*) were calculated using data across three separate days (*n* = 12) with the following equations described below. In order to calculate anisotropy from parallel and perpendicular fluorescence intensity values, data are fit to the equation (48) anisotropy =(Ipar − Iper)/(Ipar + 2Iper), where Ipar equals parallel fluorescence intensity and Iper equals perpendicular fluorescence intensity. In order to calculate *K_D_* under nonsaturating ligand conditions, data are fit to the quadratic model (49) *A* = (*A*_max_ − *A*_min_) (2*D_t_*)[(*P_t_* + *D_t_* + *K_D_*) − √(*P_t_* + *D_t_* + *K_D_*)^2^ − (4*P_t_* × *D_t_*)] + *A*_min_, where *D_t_* is the total amount of labeled ssDNA, *P_t_* is the total amount of Rep protein, *K_D_* is the dissociation constant, *A*_max_ is the anisotropy ceiling, and *A*_min_ is the anisotropy floor. MBP-dDCV fusions were used to increase the change in anisotropy signal caused by fluorescently labeled substrate binding via an increased molecular weight and thus slower rotation in solution. Addition of the N-terminal MBP fusion had only a modest impact on the dDCV Rep’s affinity for its substrate (Fig. S1).

Sequence specificity of DCV Rep for ssDNA substrates was assessed via competition anisotropy assays in a similar manner to reference 23. The ability of a mutagenized unlabeled competitor oligonucleotide to inhibit cognate Ori binding was measured in triplicate by titration of competitor via 1:2 serial dilutions starting at 10 μM into a constant concentration of 40 nM MBP-dDCV, 50 nM FAM-labeled cognate Ori, and either 5 μM MnCl_2_ or 500 μM MgCl_2_ in the same reaction buffer used in affinity experiments. IC_50_ values were calculated via fitting to the equation *Y* = bottom + (top − bottom)/[1 + (*X*/IC_50_)], where *X* is the concentration of competitor oligonucleotide, *Y* is the response value reported in anisotropy, top and bottom are the maximum and minimum concentrations of competitor, respectively, and IC_50_ is the concentration of competitor at which half of maximal inhibition is achieved.

All data were collected using a BioTek Synergy Neo2 hybrid multimode plate reader with an excitation wavelength of 485 nm (20 nm bandwidth). Emissions of 528 nm were measured at ambient temperature. Measurements were fit and plotted in GraphPad Prism according to the above-described equations.

### Molecular beacon cleavage assays.

The cleavage rate of recombinant DCV Rep protein was evaluated with a molecular beacon assay using an ssDNA probe harboring the cognate DCV Ori sequence labeled containing a 5′ IowaBlack-FQ quencher and 3′ fluorescein (D*-ori*-QF; Integrated DNA Technologies). All cleavage reactions were performed in a cleavage buffer containing 50 mM HEPES, pH 8, 1 mg/mL BSA, 0.05% Tween 20, 1 mM DTT, 50 mM to 300 mM NaCl, and 0.005 to 0.5 mM MnCl_2_ or MgCl_2_. DCV Rep was diluted with the cleavage buffer to 2 μM, and D*-ori*-QF was diluted to 200 nM in an identical buffer. In a black 96-well plate, 100 μL of D*-ori*-QF was added to each well. Using an 8-channel pipette, 100 μL of DCV was simultaneously added and manually mixed into the 8 wells containing D-ori-QF with the specified buffer. After an approximately 5-s delay, fluorescent signal was collected by a Synergy Neo2 hybrid multimode plate reader (Bio-Tek) using the monochromator function with an excitation wavelength of 485 ± 20 nm and emission collected using a 528- ± 20-nm bandpass filter at room temperature. Emission values for all 8 reactions were collected every 2 s for 20 min. At a minimum, three independent replicates were collected for each buffer condition or DNA sequence. Individual data sets were fit using GraphPad Prism to either a one- or two-phase exponential decay model to calculate and average fast- and slow-rate constants (*k*_fast_ and *k*_slow_, respectively) and respective standard errors.

### Molecular beacon reversal assays.

The extent of substrate reversal/reunion by recombinant DCV Rep protein was evaluated with a molecular beacon assay similar to that of the cleavage reaction. We prereacted 2 μM DCV Rep protein with 200 μM 3′ fluorescein-labeled Ori-derived oligonucleotide at ambient temperature for 30 min in the same cleavage buffer described in the molecular beacon cleavage assay but with either 1 mM MgCl_2_ or 1 mM MnCl_2_. In a black 96-well plate, 100 μL of this cleavage reaction was added to each of eight wells. Using an 8-channel pipette, 100 μL of the specified amount of 5′ quencher-labeled Ori-derived reunion substrate in an identical buffer was simultaneously added and manually mixed into the 8 wells containing DCV-fluorescein-Ori conjugates. In the case of EDTA reactions, the reunion solution contained a 1-mM concentration of EDTA to chelate all divalent cations from the cleavage step. After an approximately 5-s delay, fluorescent signal was collected by a Synergy Neo2 hybrid multimode plate reader using the monochromator function with an excitation wavelength of 485 ± 20 nm, and emission was collected using a 528- ± 20-nm bandpass filter at room temperature. Emission values for all 8 reactions were collected every 2 s for 20 min. At a minimum, two independent replicates were collected for each of the conditions or DNA sequences.

### WC- and rWC-HUH-seq ssDNA library cleavage, reversal, preparation, and sequencing.

For wcHUH-seq, a 60-nt ssDNA library composed of a central DCV Ori sequence with its WC base pair-forming −4 and +1 bases mutagenized (TATNATT*NC) flanked by conserved regions containing primer binding sites was purchased from IDT. DCV Rep cleavage of the library was carried out in triplicate in reactions of 3 μM DCV Rep and 3 μM library in 50 mM HEPES, pH 8.0, 50 mM NaCl, 1 mg/mL BSA, 0.05% Tween 20, and 1 mM either MnCl_2_ or MgCl_2_ for 1 h at ambient temperatures. The reactions were then heat inactivated by boiling at 95°C for 5 min.

For rwcHUH-seq, four separate 47-nt ssDNA oligonucleotides composed of a 5′-end DCV Ori sequence with its WC base pair-forming −4 and +1 bases mutagenized to one of the four potential pairings (i.e., A·T, T·A, G·C, or C·G) and a 3′ conserved region containing a primer binding site were individually prereacted at concentrations of 3 μM with 3 μM DCV in the buffer described above with 1 mM either MnCl_2_ or MgCl_2_ for 1 h at ambient temperatures. The efficiency of each of the four reactions across both cation conditions was gauged via SDS-PAGE analysis (Fig. S1), and the reactions were mixed to achieve an equal amount of each of the four +1N DCV conjugates in a volume of 25 μL at a concentration of roughly 1.2 μM (i.e., 30 pmol of +1N DCV conjugates in solution, 7.5 pmol of each construct). Following this, a 5-μL volume of a 25× mole ratio (i.e., 750 pmol) of a reunion library composed of a 5′ conserved region containing a primer binding site and a 3′ reunion Ori sequence with its −4 base mutagenized (TATNATT) in an identical buffer was mixed into the +1N DCV conjugates, diluted to 1 μM, and incubated at ambient temperatures for 1 h. The reunion reaction was then heat inactivated by boiling at 95°C for 5 min.

Following heat inactivation, 10-fold dilutions of reference, cleavage, or reunion reaction were amplified individually using 0.5 μM TruGrade high-performance liquid chromatography (HPLC) purified primers from IDT containing Nextera adapters and spacer regions to extend the amplicon length with 2× CloneAmp HiFi PCR premix (TaKaRa) for 30 cycles. The resulting amplicon was a 150-bp double-stranded DNA (dsDNA) product that was then gel purified (NucleoSpin gel and PCR clean-up kit; Macherey-Nagel). All samples were then sent individually for next-generation sequencing (Amplicon-EZ; Genewiz).

### wc- and rwcHUH-seq data analysis.

Raw NGS data were processed using the Biopython package (50). Only the forward of paired-end reads were used in data analysis due to the generally low quality of the reverse reads. Reads were selected for processing based on length and truncated to extract only the 10-nt Ori sequences (TATNATTNCC). For wcHUH-seq, mean read counts for each of the 16 unique sequences were calculated for experimental and reference libraries across three replicates. Experimental libraries were reacted with DCV and either 1 mM MnCl_2_ or MgCl_2_ in three independent replicates, whereas reference libraries were sequenced without reaction to determine the baseline enrichment for each of the 16 unique substrates in solution. Mean read counts for each experimental condition were compared against the reference to calculate percent reductions (reference − experimental/reference) for each of the 16 unique sequences in the Mn^2+^ and Mg^2+^ cleavage reaction conditions. The calculations were used to generate heatmaps depicting percent reduction with respect to reference for each substrate base combination using GraphPad Prism. For rwcHUH-seq, mean read counts for each of the 16 possible unique sequences were calculated and used to determine the percentage that each of the 16 base combinations contributes to the total number of reads in the Mn^2+^ and Mg^2+^ reunion reaction conditions. The calculations were used to generate heatmaps depicting percent composition reversed for each substrate base combination using GraphPad Prism.

### Ori-Seek genomic analysis.

Ori-Seek is a Python script heavily inspired by StemLoop-Finder (30) that uses the Biopython (50) and ViennaRNA 2.0 (32) packages to search for the origin of replication in a CRESS-DNA viral genome based on predefined nonanucleotide search terms and their ability to form stem-loop structures. It reads a combined FASTA file containing all available complete CRESS-DNA viral genomes downloaded from NCBI Virus (31) and sequentially searches the provided genomes for the predefined search terms. Once a genome has an Ori predicted, it is excluded from future searches with the remaining nonanucleotide search terms. The script outputs a tab-delimited file containing accession numbers and genome descriptions, putative Ori sequences, putative secondary structures, and the structure’s minimum free energy for each prediction grouped by search term. All noncognate WC base pairings, non-WC base pairings, and less common Ori sequences predicted by Ori-Seek were confirmed manually and had their secondary structures predicted by the mfold web server (51). Genomes that returned false predictions upon manual confirmation were listed and excluded from the final search and data export.

### Data availability.

Cocrystal structure coordinates and structure factors of DCV^Y91F^ plus Mn^2+^ plus 10-mer and DCV^Y91F^ plus 10-mer were deposited in the Protein Data Bank with the accession nos. 7KII and 7KIJ, respectively. Ori-Seek Ori sequence predictions are available in the supplemental material and on our lab website (www.mechanosome.org) under the resources section. All code used in the preparation of the manuscript and their associated files is available on both GitHub (https://github.com/AdamTSmiley) and our lab website (linked above) under the resources section.
