# Supplementary figures and images for: Watson-Crick Base-Pairing Requirements for ssDNA Recognition and Processing in Replication-Initiating HUH Endonucleases
Source: mBio. 2022 Dec 21;14(1):e02587-22. doi: 10.1128/mbio.02587-22 (PMC9973303; doi:10.1128/mbio.02587-22)

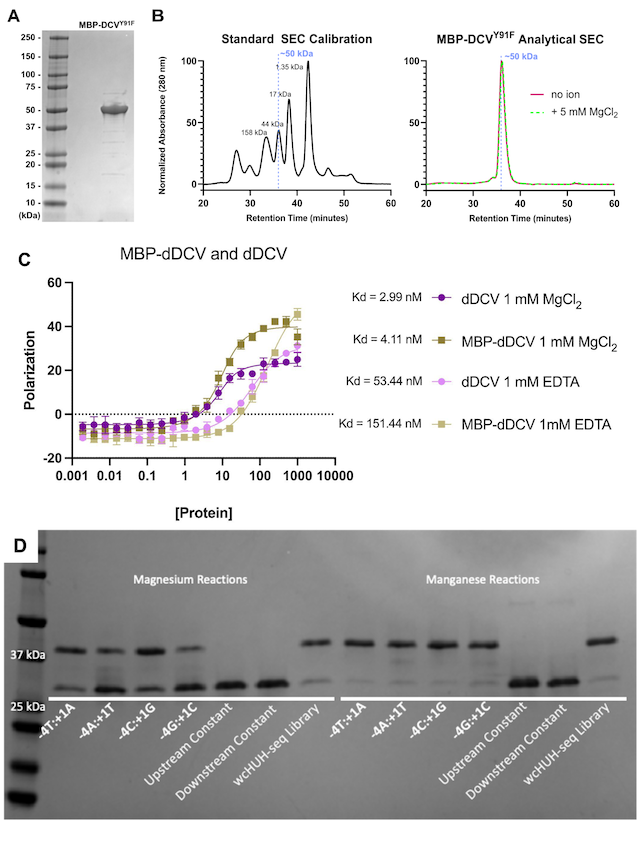

Supplement: FIG S1 [file mbio.02587-22-s0001.tif]

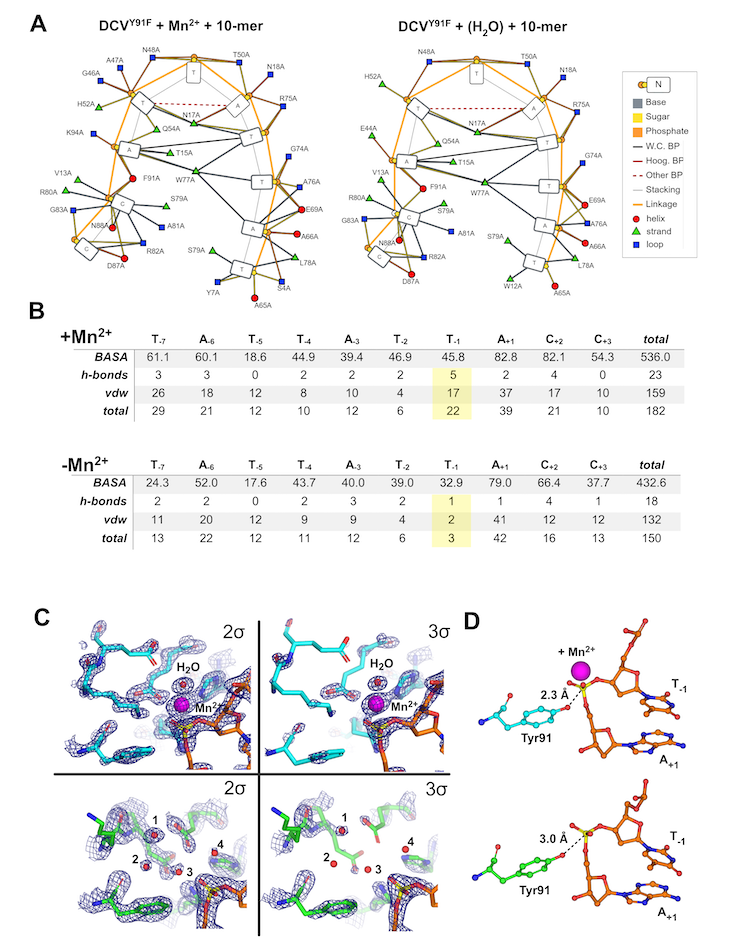

Supplement: FIG S2 [file mbio.02587-22-s0002.tif]

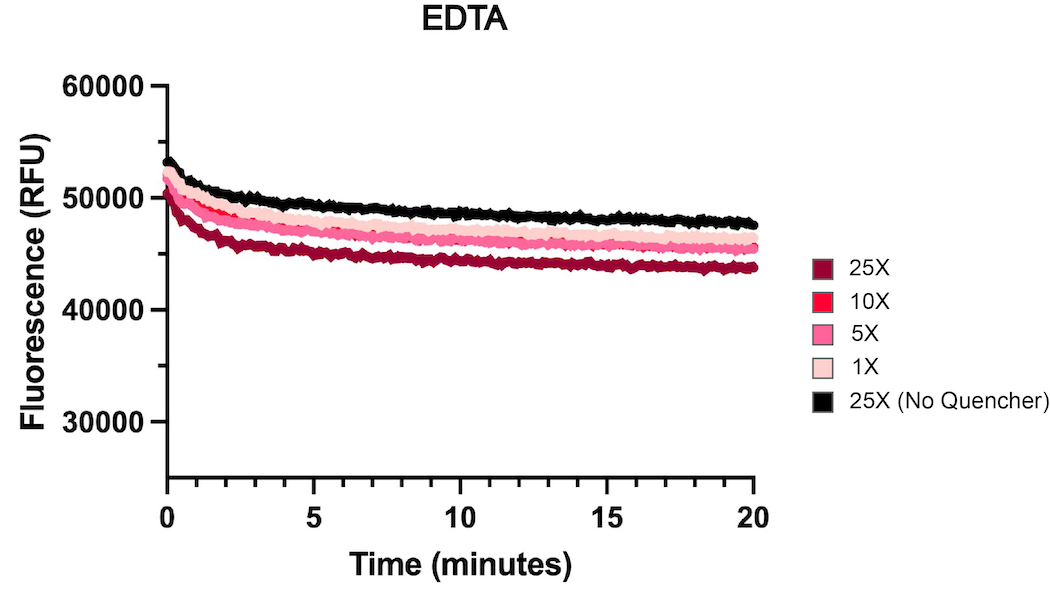

Supplement: FIG S3 [file mbio.02587-22-s0003.tif]
