# Supplementary material for: Watson-Crick Base-Pairing Requirements for ssDNA Recognition and Processing in Replication-Initiating HUH Endonucleases
Source: mBio. 2022 Dec 21;14(1):e02587-22. doi: 10.1128/mbio.02587-22 (PMC9973303; doi:10.1128/mbio.02587-22)
Supplement: TABLE S1 [file mbio.02587-22-s0004.docx]

| **Table S1** |  |  |
| --- | --- | --- |
| Ca (mM) | Kd (nM) | n = 2 |
| 1 | 135.8 ± 82.5 |  |
| 0.5 | 142.0 ± 38.5 |  |
| 0.1 | 192.45 ± 47.7 |  |
| 0.05 | 218.7 ± 48.5 |  |
|  |  |  |
| 0.5 mM Mg |  |  |
| NaCl | Kd (nM) | n = 1 |
| 200 | 1520 |  |
| 150 | 540 |  |
| 100 | 174.7 |  |
| 50 | 37.3 |  |
|  |  |  |
| 0.005 mM MnCl |  |  |
| NaCl | Kd (nM) | n = 2 |
| 200 | 27.3 ± 0.4 |  |
| 150 | 14.7 ± 4.7 |  |
| 100 | 3.15 ± 0.24 |  |
| 50 | 0.69 ± 0.35 |  |
