# Supplementary material for: Watson-Crick Base-Pairing Requirements for ssDNA Recognition and Processing in Replication-Initiating HUH Endonucleases
Source: mBio. 2022 Dec 21;14(1):e02587-22. doi: 10.1128/mbio.02587-22 (PMC9973303; doi:10.1128/mbio.02587-22)
Supplement: TABLE S2 [file mbio.02587-22-s0005.docx]

| **Table S2** |  |  |
| --- | --- | --- |
|  | **dDCV + Mn + 10-mer** | **dDCV + 10-mer** |
|  | **(PDB: 7KII)** | **(PDB: 7KIJ)** |
| Wavelength (Å) | 0.9791 | 0.9791 |
| Resolution Range (Å) | 48.56 - 1.3 (1.346 - 1.3) | 54.65 - 1.69 (1.751 - 1.69) |
| Space Group | I222 | P6_1_ 22 |
| Unit Cell (Å) | 55.417 66.343 71.259 90° 90° 90° | 102.986 102.986 138.294 90° 90° 120° |
| Total Reflections | 148935 (13772) | 425382 (40672) |
| Unique Reflections | 32195 (3197) | 48935 (4791) |
| Multiplicity | 4.6 (4.3) | 8.7 (8.5) |
| Completeness (%) | 98.42 (99.35) | 99.84 (99.69) |
| Mean I/sigma(I) | 12.24 (2.18) | 32.89 (2.28) |
| Wilson B-factor | 14.46 | 28.27 |
| R-merge | 0.053 (0.368) | 0.036 (0.825) |
| Rmeas | 0.059 (0.419) | 0.037 (0.878) |
| R-pim | 0.025 (0.196) | 0.013 (0.292) |
| CC1/2 | 0.998 (0.943) | 1 (0.784) |
| CC* | 1 (0.985) | 1 (0.938) |
| Reflections used in refinement | 32141 (3192) | 48924 (4788) |
| Reflections used for R-free | 1636 (163) | 2401 (251) |
| R-work | 0.149 (0.275) | 0.153 (0.300) |
| R-free | 0.186 (0.297) | 0.178 (0.248) |
| CC(work) | 0.974 (0.948) | 0.967 (0.913) |
| cc(free) | 0.951 (0.905) | 0.967 (0.868) |
| Number of non-hydrogen atoms | 1234 | 2258 |
| Macromolecules | 1038 | 1950 |
| Ligands | 8 | 27 |
| Solvent | 188 | 281 |
| Protein residues | 103 | 198 |
| RMS(bonds) | 0.006 | 0.007 |
| RMS(angles) | 1.01 | 0.99 |
| Ramachandran favored (%) | 100 | 98.45 |
| Ramachandran allowed (%) | 0 | 1.55 |
| Ramachandran outliers (%) | 0 | 0 |
| Rotamer outliers (%) | 0 | 0 |
| Clashscore | 0 | 1.63 |
| Average B-factor | 23.2 | 35.6 |
| Macromolecules | 20.73 | 33.44 |
| Ligands | 65.81 | 64.59 |
| Solvent | 35.05 | 47.79 |
