# Supplementary material for: Watson-Crick Base-Pairing Requirements for ssDNA Recognition and Processing in Replication-Initiating HUH Endonucleases
Source: mBio. 2022 Dec 21;14(1):e02587-22. doi: 10.1128/mbio.02587-22 (PMC9973303; doi:10.1128/mbio.02587-22)
Supplement: TABLE S3 [file mbio.02587-22-s0006.docx]

| **Table S3** |  |  |
| --- | --- | --- |
|  | **Mn (0.005 mM)** | **Mg (0.5 mM)** |
| **Sequence** | **IC50 (µM)** | |
| -5T --> A | 0.49 | 2.5 |
| -5T --> C | 0.24 | 3.2 |
| -5T --> G | 0.24 | 5.6 |
| +2C --> A | ND | ND |
| +2C --> T | ND | ND |
| +2C --> G | ND | ND |
| -7T --> G | 0.22 | 5.3 |
| -6A --> C | ND | ND |
| -4T --> G | ND | ND |
| -3A --> C | 0.097 | 5 |
| -2T --> G | 0.35 | 15 |
| -1T --> G | ND | ND |
| +1A --> C | ND | ND |
| +3C --> A | 0.15 | 0.8 |
| **Sequence** | **IC50 (µM)** | |
| **WC** |  |  |
| cognate | 0.057 ± 0.039 (n = 10) | 0.27 ± 0.05 (n = 10) |
| -4T +1T | ND | ND |
| -4T +1G | ND | ND |
| -4T +1C | ND | ND |
| -4A +1T | 9.96 | 28.2 |
| -4A +1A | ND | ND |
| -4A +1G | ND | ND |
| -4A +1C | ND | ND |
|  |  |  |
| -4G +1A | ND | ND |
| -4G +1G | ND | ND |
| -4G +1C | 7.36 | 3.6 |
| -4C +1T | ND | ND |
| -4C +1A | ND | ND |
| -4C +1G | ND | ND |
| -4C +1C | ND | ND |
