# Supplementary material for: Watson-Crick Base-Pairing Requirements for ssDNA Recognition and Processing in Replication-Initiating HUH Endonucleases
Source: mBio. 2022 Dec 21;14(1):e02587-22. doi: 10.1128/mbio.02587-22 (PMC9973303; doi:10.1128/mbio.02587-22)
Supplement: TABLE S4 [file mbio.02587-22-s0007.docx]

| **Table S4** |  |  |  |  |  |  |
| --- | --- | --- | --- | --- | --- | --- |
|  |  | Mn2+ | |  | Mg2+ | |
| [ion] |  | *kfast (min-1)* | *kslow (min-1)* |  | *kfast (min-1)* | *kslow (min-1)* |
| 0.5 mM |  | 7.42 ± 1.66 | 0.09 ± 0.04 |  | 1.67 ± 0.24 | 0.22 ± 0.12 |
| 0.1 mM |  | 2.29 ± 0.16 | 0.10 ± 0.08 |  | 0.65 ± 0.02 | 0.11 ± 0.01 |
| 0.025 mM |  | 1.36 ± 0.09 | 0.14 ± 0.09 |  | 0.36 ± 0.02 | 0.09 ± 0.07 |
| 0.005 mM |  | 1.02 ± 0.03 | 0.11 ± 0.04 |  | 0.22 ± 0.003 | - |
| 0.5 mM |  | Mn2+ | |  | Mg2+ | |
| [NaCl] |  | *kfast* | *kslow* |  | *kfast* | *kslow* |
| 50 mM |  | 7.42 ± 1.66 | 0.09 ± 0.04 |  | 1.67 ± 0.24 | 0.22 ± 0.12 |
| 100 mM |  | 7.63 ± 0.69 | 0.17 ± 0.03 |  | 0.95 ± 0.05 | 0.23 ± 0.02 |
| 150 mM |  | 4.65 ± 0.47 | 0.12 ± 0.11 |  | 0.46 ± 0.04 | 0.10 ± 0.06 |
| 300 mM |  | 1.07 ± 0.23 | 0.17 ± 0.23 |  | 0.099 ± 0.003 | - |
