# Supplementary material for: Watson-Crick Base-Pairing Requirements for ssDNA Recognition and Processing in Replication-Initiating HUH Endonucleases
Source: mBio. 2022 Dec 21;14(1):e02587-22. doi: 10.1128/mbio.02587-22 (PMC9973303; doi:10.1128/mbio.02587-22)
Supplement: TABLE S5 [file mbio.02587-22-s0008.docx]

| **Table S5** |  |  |  |  |  |  |  |
| --- | --- | --- | --- | --- | --- | --- | --- |
| **sequence (5' --> 3')** | **name** |  | ***kfast* (min-1)** | ***kslow* (min-1)** |  | ***kfast* (min-1)** | ***kslow* (min-1)** |
| **T A T T A T T * A C** | **D-*ori*** |  | 7.42 ± 1.66 | 0.09 ± 0.04 |  | 1.67 ± 0.24 | 0.22 ± 0.12 |
| **• • A • • • • * • •** | **-5A** |  | 12.72 ± 1.84 | 0.06 ± 0.01 |  | 0.88 ± 0.05 | 0.06 ± 0.01 |
| **• • C • • • • * • •** | **-5C** |  | 7.64 ± 0.73 | 0.10 ± 0.01 |  | 0.67 ± 0.04 | 0.13 ± 0.01 |
| **• • G • • • • * • •** | **-5G** |  | 5.33 ± 0.81 | 0.11 ± 0.01 |  | 0.27 ± 0.08 | 0.06 ± 0.02 |
