# Supplementary material for: Watson-Crick Base-Pairing Requirements for ssDNA Recognition and Processing in Replication-Initiating HUH Endonucleases
Source: mBio. 2022 Dec 21;14(1):e02587-22. doi: 10.1128/mbio.02587-22 (PMC9973303; doi:10.1128/mbio.02587-22)
Supplement: TABLE S7 [file mbio.02587-22-s0010.docx]

| **Table S7** |  |  |  |
| --- | --- | --- | --- |
| **Non-WC Ori Genomes** | **Putative Ori** | **WC Ori Genomes** | **Putative Ori** |
| MW700156.1 \|Digitaria streak virus isolate ISV 2001 clone 26, complete genome | TAA**A**ATT**A**C | MW700085.1 \|Digitaria streak virus isolate ISV 1990, complete genome | TAA**T**ATT**A**C |
| KX533467.1 \|Turnip curly top virus isolate TCTV_IR_CZ1_2013, complete genome | TAA**T**ATT**C**C | MF536416.1 \|Turnip curly top virus isolate IR:Lap:L2-7:Jim:13, complete genome | TAA**T**ATT**A**C |
| KP641674.1 \|Tomato leaf curl New Delhi virus segment DNA B, complete sequence | TAA**C**ATT**A**C | MG715488.1 \|Tomato yellow leaf curl virus isolate ES-Mlg-TY16-Tom-2017, complete genome | TAA**T**ATT**A**C |
| KM595218.1 \|Squash leaf curl virus isolate PA1-J245, complete genome | TAA**T**ATT**C**C | KM595239.1 \|Squash leaf curl virus isolate PA3-T7, complete genome | TAA**T**ATT**A**C |
| JX915744.1 \|Jatropha curcas mosaic virus isolate SG segment DNA-B, complete sequence | TAA**T**ATT**C**C | KF998097.1 \|Jatropha mosaic virus isolate Fl-USA segment DNA-A, complete sequence | TAA**T**ATT**A**C |
| JQ707943.1 \|Beet curly top Iran virus isolate IR:Kam:B24K:Sug:08, complete genome | TAA**C**ATT**C**C | JQ707938.1 \|Beet curly top Iran virus isolate IR:Yaz:B15P:Sug:06, complete genome | TAA**G**ATT**C**C |
| JQ647477.1 \|Wheat dwarf virus isolate QHLD10-1, complete genome | TAA**A**ATT**A**C | JQ361910.1 \|Wheat dwarf India virus, complete genome | TAA**T**ATT**A**C |
| FJ176236.1 \|Bhendi yellow vein mosaic virus [2005:Pandaralli:India] segment DNA-A, complete sequence | TAA**C**ATT**A**C | GU112079.1 \|Bhendi yellow vein mosaic virus [India:Pandarahalli:OY167:2006] segment DNA-A, complete sequence | TAA**T**ATT**A**C |
| EF107520.1 \|Tomato yellow leaf curl virus isolate TYLCV-Nob, complete genome | TAA**T**ATT**T**C | EF101929.1 \|Tomato yellow leaf curl virus - [Tunisia], complete sequence | TAA**T**ATT**A**C |
| MW117136.1 \|Porcine circovirus 2 isolate HeNA7, complete genome | GAT**A**ATT**A**C | AY969004.1 \|Porcine circovirus 2 isolate Henan, complete genome | AAG**T**ATT**A**C |
| KT180275.1 \|Banana bunchy top virus isolate Trp-GO1 segment DNA-S, complete sequence | TAT**T**ATT**T**C | MT433364.1 \|Banana bunchy top virus isolate GM_72006 segment DNA S, complete sequence | TAT**T**ATT**A**C |
| MN709511.1 \|Canine circovirus isolate CQ82, complete genome | TAG**T**ATT**G**C | NC_020904.1 \|Canine circovirus isolate UCD1-1698, complete genome | TAG**T**ATT**A**C |
| KJ866054.1 \|Beak and feather disease virus isolate BFDV_AUS_MRP_14_1195_001, complete genome | TAG**T**ATT**C**C | KM887916.1 \|Beak and feather disease virus isolate BFDV_AUS_RBL_2010_340, complete genome | TAG**T**ATT**A**C |
