# Supplementary material for: Watson-Crick Base-Pairing Requirements for ssDNA Recognition and Processing in Replication-Initiating HUH Endonucleases
Source: mBio. 2022 Dec 21;14(1):e02587-22. doi: 10.1128/mbio.02587-22 (PMC9973303; doi:10.1128/mbio.02587-22)
Supplement: TABLE S6 [file mbio.02587-22-s0009.docx]

| **Table S6** |  |  |  |  |  |
| --- | --- | --- | --- | --- | --- |
| **WC-Containing Oris** | | | | | |
| **Search Term** | | | | | |
| **TAANATTNC** | | **NATNATTNC** | | **NAGNATTNC** | |
| pOri | Count | pOri | Count | pOri | Count |
| TAA**T**ATT**A**C | 13,420 | TAT**T**ATT**A**C | 1,755 | AAG**T**ATT**A**C | 3,105 |
| TAA**G**ATT**C**C | 100 | CAT**T**ATT**A**C | 31 | TAG**T**ATT**A**C | 2,926 |
| TAA**C**ATT**G**C | 3 | AAT**T**ATT**A**C | 6 | CAG**T**ATT**A**C | 162 |
| TAA**A**ATT**T**C | 1 | GAT**T**ATT**A**C | 2 | GAG**T**ATT**A**C | 26 |
| - | - | TAT**A**ATT**T**C | 1 | TAG**A**ATT**T**C | 5 |
| **WC-Lacking Oris** | | | | | |
| **Search Term** | | | | | |
| **TAANATTNC** | | **NATNATTNC** | | **NAGNATTNC** | |
| pOri | Count | pOri | Count | pOri | Count |
| TAA**A**ATT**A**C | 3 | TAT**A**ATT**A**C | 1 | TAG**T**ATT**G**C | 1 |
| TAA**T**ATT**C**C | 6 | AAT**A**ATT**A**C | 3 | TAG**A**ATT**A**C | 1 |
| TAA**C**ATT**A**C | 2 | GAT**A**ATT**A**C | 1 | TAG**T**ATT**C**C | 2 |
| TAA**C**ATT**C**C | 1 | TAT**T**ATT**T**C | 1 | - | - |
| TAA**T**ATT**T**C | 1 | - | - | - | - |
